# Transfer Entropy as a Measure of Brain Connectivity: A Critical Analysis With the Help of Neural Mass Models

**DOI:** 10.3389/fncom.2020.00045

**Published:** 2020-06-05

**Authors:** Mauro Ursino, Giulia Ricci, Elisa Magosso

**Affiliations:** Department of Electrical, Electronic and Information Engineering, University of Bologna, Cesena, Italy

**Keywords:** bivariate transfer entropy, connectivity, neural mass models, excitatory and inhibitory synapses, information transfer, causality, non-linear neural phenomena, Trentool software

## Abstract

**Objective:** Assessing brain connectivity from electrophysiological signals is of great relevance in neuroscience, but results are still debated and depend crucially on how connectivity is defined and on mathematical instruments utilized. Aim of this work is to assess the capacity of bivariate Transfer Entropy (TE) to evaluate connectivity, using data generated from simple neural mass models of connected Regions of Interest (ROIs).

**Approach:** Signals simulating mean field potentials were generated assuming two, three or four ROIs, connected via excitatory or by-synaptic inhibitory links. We investigated whether the presence of a statistically significant connection can be detected and if connection strength can be quantified.

**Main Results:** Results suggest that TE can reliably estimate the strength of connectivity if neural populations work in their linear regions, and if the epoch lengths are longer than 10 s. In case of multivariate networks, some spurious connections can emerge (i.e., a statistically significant TE even in the absence of a true connection); however, quite a good correlation between TE and synaptic strength is still preserved. Moreover, TE appears more robust for distal regions (longer delays) compared with proximal regions (smaller delays): an approximate a priori knowledge on this delay can improve the procedure. Finally, non-linear phenomena affect the assessment of connectivity, since they may significantly reduce TE estimation: information transmission between two ROIs may be weak, due to non-linear phenomena, even if a strong causal connection is present.

**Significance:** Changes in functional connectivity during different tasks or brain conditions, might not always reflect a true change in the connecting network, but rather a change in information transmission. A limitation of the work is the use of bivariate TE. In perspective, the use of multivariate TE can improve estimation and reduce some of the problems encountered in the present study.

## Introduction

Cognitive phenomena originate from the interaction among several mutually interconnected, specialized brain regions, which exchange information via long range synapses. Consequently, the problem of assessing brain connectivity during different cognitive tasks is playing a crucial role in neuroscience nowadays, not only to understand mechanisms at the basis of normal cognitive functions, but also to identify alterations in pathological states. Connectivity is often estimated from fMRI neuroimaging techniques (Horwitz, 2003; Friston, 2009; van den Heuvel and Hulshoff Pol, 2010). However, thanks to their higher temporal dynamics, electrophysiological data, obtained from electro- or magneto-encephalography, joined with methods for cortical source localization (Koenig et al., 2005; Astolfi et al., 2007; Sakkalis, 2011; Rossini et al., 2019) are receiving increasing attention.

The problem of assessing connectivity from data, however, is a difficult one, since the concept of connectivity has ambiguous definitions (see Horwitz, 2003) and results depend crucially on how connectivity is defined and on the mathematical instruments utilized.

Although there are different ways to define connectivity, in the following we will refer to *functional connectivity* (FC) defined as “the statistical dependence or mutual information between two neuronal systems” (Friston et al., 2013). A distinct definition of connectivity, stronger than FC (see Valdes-Sosa et al., 2011; Friston et al., 2013), is *effective connectivity*: it refers to the influence that one neural system exerts on another and is based on an explicit model of causal inference, usually expressed in terms of differential equations. The most popular method to evaluate effective connectivity is dynamical causal modeling (DCM). DCM assumes that the signals are produced by a state space model (see Table 1 in Valdes-Sosa et al., 2011, for a list of possible equations used in recent papers). However, this framework requires strong a priori knowledge about the input to the system and the connectivity network. To overcome this limitation, the more suitable network is often chosen among various possible alternatives using Bayesian selection methods (Penny et al., 2004).

However, despite these dichotomous definitions, the fundamental interest in all FC research is still “understanding the casual relationship among neural entities,” as stressed by Reid et al. (2019) recently. Although the kind of causal inference that can be inferred with FC methods is limited and only indirect, several FC measures can provide some useful information regard to causality (recent assessment papers are Wang et al., 2014; Bastos and Schoffelen, 2015; Reid et al., 2019). Indeed, among the different ways to calculate FC, some of them are based on the concept of causality (although without using state-space models), as originally introduced by Wiener (1956) and subsequently by Granger (1980). According to their definition, we can say that a temporal series X has a causal influence on a second temporal series Y if the prediction on the future of Y is improved by knowledge on the past of X. Interestingly, the technical links between Granger causality and DCM have also been recently incorporated in the state space framework (Bajaj et al., 2016) with reference to functional magnetic data in resting states. Results of these authors indicate a qualitative consistency between Granger causality and DCM, and show that both can be used to estimate directed functional and effective connectivity from fMRI measurements in a reliable way.

One of the most promising method to infer FC from data is Transfer entropy (TE). TE implements the causal principle expressed above within the framework of information theory, by using conditional probabilities (Schreiber, 2000; Vicente et al., 2011, for more details): if a signal X has a causal influence on a signal Y, then the probability of Y conditioned on its past is different from the probability of Y conditioned on both its past and the past of X. The same idea can be expressed observing that entropy on the present measurement of Y is reduced if knowledge of the past of X is added to knowledge of the past of Y. A great advantage of TE compared with the other methods is that it does not require any prior assumption on data generation (i.e., it is model-free).

For this reason, TE is largely used in neuroscience today to assess connectivity from EEG/MEG data sets in conditions lacking any prior assumption. Also some variants of TE (such as Phase Transfer Entropy, Lobier et al., 2014) have been proposed recently.

Nevertheless, the use of TE to assess connectivity may also exhibit some drawbacks, besides definite advantages. First, as recognized in several papers (Schindler et al., 2007; Vicente et al., 2011; Wollstadt et al., 2014), estimation of TE from data can be affected by various elements of the estimation procedure; among the others: the embedding dimension and the delay in the reconstruction of the state space, the quantity of data samples available, the method adopted to estimate high-dimensional conditional probabilities.

Second, it is unclear how much TE is affected by spurious information, such as that arising from shared inputs or from a cascade among several populations, or due to a redundancy in the population processing (Wibral et al., 2014). To reduce the previous aspects, multivariate TE methods have also been proposed recently (Montalto et al., 2014).

Third, and maybe more important, TE is not a direct measure of coupling strength, and should be used with extreme caution to measure a coupling parameter (such as the weight of synapses among two neural populations). Actually, TE measures how much information is transferred from X to Y: this concept is of the greatest value to understand how the brain performs its computation by exchanging information between different regions (see also Lizier and Prokopenko, 2010), but in some conditions may be intrinsically different from causal strength.

Once established that TE is a valid tool to investigate the computational aspects of the brain, i.e., the transfer of information between different areas, in the present study we wish to critically analyse how good it may be at estimating a biophysical coupling property too, i.e., the connection strength between Regions of Interest (ROIs). To this end, a powerful way is to challenge TE with the use of simulated data. These should mimic real neuroelectric signals (especially for what concerns their frequency content), and should be generated via biologically inspired models with assigned coupling terms among neural units.

Indeed, many such studies have been published in the last decade, to compare FC estimated values with a “true” connectivity topology incorporated in simulation models, providing quite a large set of validation information. In the following, we will first encompass a synthetic analysis of the recent literature, to point out the present major gaps and elements which, in our opinion, deserve further analysis (especially, with reference to TE). Then, the aim of this work is better delineated, as it emerges from the absences in the present literature.

## Literature Critical Review

### Summary of Previous Studies

Several studies have been performed in recent years, to compare the results obtained with the FC estimation methods, with the “true” connectivity values incorporated in simulation models (used as a sort of “ground truth”). However, most of these studies were aimed at exploring whether FC can discover the presence of connectivity links (on/off), using receiver operating characteristic (ROC) curves. Just in a few of them, the relationship between the FC index and the connectivity strength was explored, although generally in rather a qualitative way.

Two main classes of studies will be considered in the following, depending on the simulation model adopted: those which use spiking neurons, mainly aimed at analyzing connectivity in neural cultures, and those using neurons with continuous outputs, more oriented to the analysis of connectivity among larger regions of interest. As to the studies with spiking neurons, in the following we will limit our analysis just to those which use TE. A wider approach is used for the selection of studies simulating larger cortical ROIs.

#### Studies With Spiking Neurons

Ito et al. (2011) applied the TE with multiple time delays to a network model containing 1,000 Izhikevich's neurons. They observed that their measures generally increase with synaptic weights, but there is substantial variability in the obtained results. Moreover, in their work the synaptic weights were bimodally distributed around just two values (0 and a positive one).

Garofalo et al. (2009) compared the estimation obtained with various methods (Transfer Entropy, Joint Entropy, Cross Correlation, and Mutual Information) first in a neuronal network model made up of 60 synaptically connected Izhikevic neurons, and then in cultures of neurons. In the model they also included inhibitory connections. The comparison was performed with ROC curves. Their results suggest that TE is the best method, both with the excitatory and excitatory+inhibitory models, but it recognizes also some strong indirect connections not classified as true positive. Moreover, it exhibits problems in identifying inhibitory connections.

Orlandi et al. (2014) also used realistic computational models that mimicked the characteristic bursting dynamics of neural cultures, and extended previous works by attempting the inference of both excitatory and inhibitory connectivity via TE. The quality of the reconstruction was quantified through a ROC analysis. They showed that the most difficult aspect is not the identification of a link, but rather its correct labeling (excitatory or inhibitory). Hence, they suggested a two-step analysis (for instance before and after the use of pharmacological blocking inhibitory connections).

Timme and Lapish (2018) analyzed the strength of information theory methods using both small networks of neurons and larger 1,000 neuron models of Izhikevich type (800 excitatory, 200 inhibitory). They concluded that TE can be used to measure information flow between neurons. More important, they suggested the use of partial information decomposition to move beyond pair of variables to group of variables, and found that this method can be used to break down encoding by two variables into redundant, unique and synergistic parts. The last aspect will be commented in section Discussion of the present paper.

All previous studies suggest that TE can be a powerful instrument to infer the existence of connections among neurons. However, the application of these studies is limited to cultures of about a thousand of units. Of course, the neuroelectric dynamics of an entire ROI, resulting from millions of neurons, is largely different. To study this aspect, higher levels models, with just a few states variables per ROI, are generally used, particularly neural mass models (NMMs).

#### Studies Using Neural Mass Models

A pioneering study which evaluated functional connectivity using neural mass models was performed by David et al. (2004). The authors used cross-correlation, mutual information, and synchronization indices (hence, they did not evaluate TE). For simplicity, they used a symmetric configuration and did not consider the problem of inhibitory connections among ROIs. The results suggest that each measure is sensitive to changes in neuronal coupling, with a monotonic dependence between the functional connectivity measures and the coupling parameter, and that the statistical power of each measure is an increasing monotonic function of the signal length.

Studies quite similar to the present one, although with a simpler aim, and without TE as a target, have been performed by others (Ansari-Asl et al., 2006; Wendling et al., 2009) (indeed, they used either regression methods or synchronization indices). Various models were employed to generate signals: among the others, two NMMs (but with only two populations each) connected with an excitatory coupling parameter. The authors explored the relationship between the coupling parameter and the estimated FC and observed that the regression methods exhibit good sensitivity to the coupling parameter. However, in that study the characterization of the *direction* of coupling was not dealt with, inhibitory connections were not incorporated, and the authors did not test TE accuracy.

A systematic study on the performance of various methods for FC estimation was performed by Wang et al. (2014). They compared the performance of 42 methods (including, among the others, the pairwise directed TE and the partial TE), using five different models to generate signals (including a three population NMM). Moreover, they used a connectivity structure with 5 nodes. Although this is the most complete study presently available, it limits the analysis to the performance of the connectivity estimate on an on/off basis, using ROC curves (i.e., they did not evaluate whether the estimated FC values are sensitive to the strength of the coupling parameters). Their results suggest that, for the NMM simulations, Granger causality and TE are able to recover the underlying model structure, with TE much less time consuming. However, TE failed when simulations were performed with highly non-linear (Rossler or Hénon) equations.

The previous summary highlights several important points. First, various methods do exist to infer FC from signals, each with its own virtues and limitations (but see also Reid et al., 2019). However, in many studies TE emerges as one of the most effective methods, which joins benefits of good sensitivity and efficient computation time. However, despite the excellent works performed until now, several problems are still insufficiently clarified, which justify further studies. These questions are stressed below.

First, no study analyzed carefully the relationship between the TE metrics and the connectivity strength using NMMs to simulate neuroelectrical activity of entire ROIs. Actually, neither (David et al., 2004) nor (Wendling et al., 2009) used TE in their analysis, whereas (Wang et al., 2014) (who tested TE) did not evaluate the sensitivity vs. the connection strength.

Second, despite some authors analyzed the presence of excitation + inhibition in models of spiking neurons (Garofalo et al., 2009; Orlandi et al., 2014), we are not aware of any study in which inhibition between ROIs is properly taken into account in the analysis of FC. Indeed, although long-range connections between ROIs are mediated by synapses from pyramidal neurons (hence, they are all excitatory in type) one region can inhibit another region by targeting into the population of inhibitory interneurons. In particular, it is known that lateral connections in the cortex target all population types, in different layers of a cortical column (Felleman and Van Essen, 1991; David et al., 2005). Although the role of various connections types in the propagation of brain rhythms has been carefully studied with NMMs (David and Friston, 2003; David et al., 2005; Zavaglia et al., 2008, 2010; Ursino et al., 2010; Cona et al., 2011) we are not aware of any NMM study which investigates the role of long-range inhibition on FC estimation.

Finally, and more important, several studies underline the difficulty of FC methods (and in particular, TE) to deal with strongly non-linear problems. For instance (Wang et al., 2014) observed that TE fails to find a proper connectivity topology when signals are generated with Rössler equations or Hénon systems, i.e., with strongly non-linear models. By comparing TE computed at various time lags to values computed with surrogate linearized data (Nichols et al., 2005) observed that TE is quite sensitive to the presence of non-linearity in a system. Indeed, although NMMs have been frequently used in the domain of FC assessment, to our knowledge all previous papers used these models in “quite linear conditions,” i.e., without inducing strong alterations in the working point and/or moving dynamically from linear vs. saturation activity regions. We speculate that the same model (with assigned connectivity strength) can produce largely different values of FC estimation depending on the working conditions, on noise variance and on the amplitude of the input changes.

### Objectives and Work Organization

Taking in mind the previous limitations of former works, the present study was conceived with the following major aims: (i) to analyse the relationship between the TE metrics and the strength of the connectivity parameters using NMMs, in order to assess whether changes in TE from one trial to another can be used to infer an underlying *change* in connectivity between ROIs; (ii) to study the role of synapses targeting to excitatory vs. inhibitory populations in affecting FC; (iii) to reveal how non-linearities can dramatically affect the inference of connection strength, leading to different conclusions on connectivity among regions depending on the particular working condition. This point is of value to highlight that TE is actually a powerful metrics to assess information transfer and computation in the brain, but in some cases may be different from coupling strength. We think that neither of these points has been thoroughly assessed in previous papers.

To reach these objectives, we evaluated FC with bivariate TE using data generated from simple NMMs of connected populations. In particular, the values of TE between two ROIs estimated from simulated data were compared with the strength of the coupling terms used in the model, at different values of this strength. We investigated different network topologies (with two, three or four ROIs) and the role of time delay, signal length, and changes in external input (mean value and noise variance). The latter aspect is of pivotal value to assess the role of non-linearities.

TE was estimated using Trentool, a software package implemented as a Matlab toolbox under an open source license (Lindner et al., 2011). Simulated data were generated using the model of neural masses described in Ursino et al. (2010) and Cona et al. (2011) which represents a good compromise between biological reliability and simplicity, and is able to simulate realistic spectra of neuroelectric activity in the cortex (including alpha, beta, and gamma bands). In particular, in this work the internal parameters of this model were assigned to simulate spectra with a strong component in the beta band and some component in the gamma band, as often measured in motor, premotor and supplementary motor cortices (Ursino et al., 2010; Cona et al., 2011).

The paper is structured as follows. First, the main theoretical aspects of transfer entropy are described. Subsequently, equations of the neural model are given, with parameter numerical values.

In section results, TE estimates obtained with Trentool on simulated data were used not only to test the performance of this metric in detecting the presence or absence of a connection (on/off evaluation by means of statistical tests against surrogate signals), but also to compare the TE values of the detected connections with the strength of the coupling terms in the model. Results are then critically discussed to emphasize in which conditions TE can provide reliable indications on connectivity, and in which conditions information transfer is different from connection strength. Limitations of this work (such as the use of a bivariate estimator) are also debated and lines for further work delineated.

## Transfer Entropy: Theoretical and Practical Aspects

In the following, we first summarize the main theoretical aspects of transfer entropy, as a model-free method to estimate connectivity. Then, some practical issues of the estimation procedure adopted by Trentool are discussed.

### General Theory

Throughout this section, we will use a lower case letter to denote a single (scalar) variable, and an upper case letter to denote a vector. Moreover, we will use the boldface to represent a random variable (or a random vector) and no-bold to represent the realization of these variables during the experiment.

Let us consider a discrete random variable **x**, with realization *x* ∈ *S*_*x*_ and probability distribution *p*(*x*) over its outcomes. The amount of *information* gained by observation of the event *x* is

$$\begin{array}{l} h\left(x\right)={\text{log}}_{2}\frac{1}{p\left(x\right)}=-{\text{log}}_{2}\;p\left(x\right) \end{array}$$

For instance, if a discrete event has probability *p*(*x*) = 1/8 = 2^−3^, its realization provides three bits of information.

Shannon entropy of the random variable **x** is computed as the average value of the information over all possible realizations of *x*, i.e.,

$$\begin{array}{l} S\left(\text{x}\right)=\underset{x\in S_{x}}{\sum }p\left(x\right){\text{ log}}_{2}\frac{1}{p\left(x\right)}=-\underset{x\in S_{x}}{\sum }p\left(x\right){\text{log}}_{2}\;p\left(x\right) \end{array}$$

The same definition of Shannon entropy, of course, can be applied in case of conditional probability. Let us assume that we observe the outcome of a discrete random variable **y** (with probability distribution *p*(*y*), and *y* ∈ *S*_*y*_) after we have already observed a realization *x* of the other random variable **x**. The amount of information gained by the observation *y* becomes

$$\begin{array}{l} h\left(y/x\right)=-{\text{log}}_{2}\;p\left(y/x\right) \end{array}$$

and, by computing the average value over all possible realization of *x* and *y*, we have

$$\begin{array}{l} S\left(\text{y}/\text{x}\right)=-\underset{x\in S_{x}}{\sum }p\left(x\right)\underset{y\in S_{y}}{\sum }p\left(y/x\right){\text{log}}_{2}\;p\left(y/x\right)\\ =-\underset{\begin{array}{c} x\in S_{x}\\ y\in S_{y} \end{array}}{\sum }p\left(x,y\right){\text{log}}_{2}\;p\left(y/x\right) \end{array}$$

Mutual information of **x** and **y** is evaluated by computing the difference between the entropy of **y**, and the conditional entropy of **y/x**. Of course, the entropy of **y** must be greater (or at lest equal) than the entropy of **y**/**x**, since observation of a realization *x* can reduce the amount of information provided by the observation *y*. The difference between the two entropies is considered as a sort of information that **x** and **y** share. Accordingly, we can define mutual information as follows

$$\begin{array}{l} I\left(\text{y},\text{x}\right)=S\left(\text{y}\right)-S\left(\text{y}/\text{x}\right) \end{array}$$

Using the Bayes theorem, one can demonstrate that

$$\begin{array}{l} I\left(\text{y},\text{x}\right)=I\left(\text{x},\text{y}\right)=S\left(\text{x}\right)-S\left(\text{x}/\text{y}\right) \end{array}$$

i.e., mutual information does not contain any directional evidence.

The same concept of mutual information can be restated assuming that both **x** and **y** are conditioned by the value of a third random variable, **z**. We obtain the conditioned mutual information

$$\begin{array}{l} I\left(\text{y},\text{x}/\text{z}\right)=S\left(\text{y}/\text{z}\right)-S\left(\text{y}/\text{x},\text{z}\right) \end{array}$$

Let us now apply the same concepts to two time series generated by two stochastic processes. From each process we can define a time-dependent random state vector (**X**^***m***^(t) and **Y**^***n***^(t), respectively), whose particular observation can be written as follows (see Takens, 1980)

$$\begin{array}{l} X^{m}\left(t\right)=\left[x\left(t\right)\;x\left(t-\Delta t\right)\;x\left(t-2\Delta t\right)\;...\;x\left(t-\left(m-1\right)\Delta t\right)\;\right] \end{array}$$

$$\begin{array}{l} Y^{n}\left(t\right)=\left[y\left(t\right)\;y\left(t-\Delta t\right)\;y\left(t-2\Delta t\right)\;...\;y\left(t-\left(n-1\right)\Delta t\right)\;\right] \end{array}$$

where *m* and *n* are the embedding dimensions, describing how many past samples are used (these are the dimensions of the so-called delay embedding space) and Δ*t* is the embedding delay. According to the previous equations, *X*^*m*^(*t*) and*Y*^*n*^(*t*) contain the present and *m-1* (or *n-1*) past samples of the random process.

Let us now consider the random variable **y**(*t*) representing a present sample of the stochastic process, conditioned by its *n* past samples; the conditional probability is p(*y*(*t*)/*Y*^*n*^(*t* − Δ*t*)) and Shannon entropy is S(*y*(*t*)/*Y*^*n*^(*t* − Δ*t*)). The idea is that, in case of causality from X to Y, the probability of **y**(*t*) conditioned by both **X**^*m*^(*t* − Δ*t*) and **Y**^*n*^(*t* − Δ*t*) should be different from the probability of **y**(*t*) conditioned by its past only. This effect can be quantified as a difference in Shannon entropy, i.e., by evaluating the additional information that the past of X provides on the present of Y. This leads to the following definition of Transfer Entropy

$$\begin{array}{l} TE\left(X\to Y\right)=I\left(\text{y}\left(t\right),{\text{X}}^{m}\left(t-\Delta t\right)/{\text{Y}}^{n}\left(t-\Delta t\right)\right)\\ =S\left(\text{y}\left(t\right)/{\text{Y}}^{n}\left(t-\Delta t\right)\right)-S\left(\text{y}\left(t\right)/{\text{X}}^{m}\left(t-\Delta t\right),{\text{Y}}^{n}\left(t-\Delta t\right)\right) \end{array}$$

TE is asymmetric and naturally incorporates direction of information transfer from X to Y.

The previous equation considers the influence that the past of Y and X can have on the present sample of Y. However, as rigorously demonstrated by Wibral et al. (2013) this equation cannot be used to express any causal relationship. In particular, in neural problems, the influence of a signal on another is often characterized by a pure delay (say *d*) which represents the time necessary for action potentials to travel along axons from the pre-synaptic region to the post-synaptic one. Assuming that the time delay can be approximated by *l* sampling periods (i.e., *d* = *l* · Δ*t*), we can use the delayed signal **X**^*m*^(*t* − *d*) = **X**^*m*^(*t* − *l* · Δ*t*) in the definition of TE instead of **X**^*m*^(*t*). In most cases, *l* is not known, and represents a parameter that should be estimated from data (see below).

We thus can write

$$\begin{array}{l} TE\left(X\to Y,l\right)=I\left(\text{y}\left(t\right),{\text{X}}^{m}\left(t-l\cdot \Delta t\right)/{\text{Y}}^{n}\left(t-\Delta t\right)\right)=\\ =S\left(\text{y}\left(t\right)/{\text{Y}}^{n}\left(t-\Delta t\right)\right)-S\left(\text{y}\left(t\right)/{\text{X}}^{m}\left(t-l\cdot \Delta t\right),{\text{Y}}^{n}\left(t-\Delta t\right)\right) \end{array}$$

Wibral et al. (2013) rigorously demonstrated that the predictive information transfer from X to Y over a time delay *d* is properly captured by this equation (aligning with Wiener's principle).

The previous equation can be rewritten as the Kullback-Leibler divergence between the two probability distributions (Schreiber, 2000)

$$\begin{array}{l} TE(X\to Y,l)=\sum_{\begin{array}{l} \;y(t)\\ \;Y^{n}(t-\Delta t)\\ \;X^{m}(t-l\cdot \Delta t) \end{array}}p(y(t),Y^{n}(t-\Delta t),X^{m}(t-l\cdot \Delta t))\\ {\text{log}}_{2}\frac{p(y(t)/Y^{n}(t-\Delta t),X^{m}(t-l\cdot \Delta t))}{p(y(t)/Y^{n}(t-\Delta t))} \end{array}$$

or also as a representation of four Shannon entropies:

$$\begin{array}{l} TE\left(X\to Y,l\right)=S\left({\text{X}}^{m}\left(t-l\cdot \Delta t\right),{\text{Y}}^{n}\left(t-\Delta t\right)\right)\\ -S\left(\text{y}\left(t\right),{\text{X}}^{m}\left(t-l\cdot \Delta t\right),{\text{Y}}^{n}\left(t-\Delta t\right)\right)+\\ +S\left(\text{y}\left(t\right),{\text{Y}}^{n}\left(t-\Delta t\right)\right)-S\left({\text{Y}}^{n}\left(t-\Delta t\right)\right) \end{array}$$

where S(**X**, **Y**) is used to denote the Shannon entropy of the joined probability of **X** and **Y**.

### Practical Aspects on TE Estimation

As it is clear from the last equation, the estimation of TE from finite data samples requires the evaluation of various joint and marginal probability distributions. This may be a difficult task, since the probability densities implicated in this equation can have a very large dimensionality (up to *n* + *m* + 1). Moreover, several parameters are not known a priori and must be estimated; in particular, the estimate of TE can be seriously affected by the choice of the embedding dimensions (*n* and m), of the sampling period (Δ*t*) and of the delay (*d* = *l*·Δ*t*). Furthermore, TE estimation can have a residual bias. To eliminate this bias, it is important to compare the TE estimated from empirical data, with that obtained from surrogate data sets. Surrogate data sets should incorporate no information transfer from X to Y, but maintain the same statistical properties as the original data. Comparison between the TE obtained from the original data and those obtained from surrogate data also allows computation of a *p*-value to test the statistical significance of the obtained TE value.

The estimates of TE from the outputs of our neural mass model (see section Transfer Entropy: Theoretical and Practical Aspects), and their statistical significance were performed using the software package Trentool (Lindner et al., 2011; Vicente et al., 2011). The same package also provides an estimation of the time delay, *l*, and of the embedding dimensions, *m* and *n*, as the values which maximize TE. In this tool, joint and marginal probability distributions are computed using a k-th nearest neighbor estimator. Furthermore, the method contains two additional parameters: the mass for the nearest-neighbor search and a correction to exclude autocorrelation effects from the density estimation. Specifically, the estimate of the bivariate TE for each model configuration was performed as follows. The same model configuration (corresponding to a specific pattern of connectivity among a few ROIs) was run 10 times creating 10 trials of signals; each trial contained the temporal patterns of the local field potentials (over a given time interval, see below) in the involved ROIs, and affected by a random noise. The 10 trials were given as input to Trentool that computed the TE values of the fed signals and of the surrogate data and provided the *p*-value (permutation test) to assess whether the TE of the simulated signals was significantly different from that of surrogate data. Furthermore, in all simulations with more than two ROIs, the results were subjected to a partial correction of spurious information flow that may be introduced by the bivariate analysis of a highly multivariate system. Namely, this correction works on cascade effects and simple common drive effects. To this end, we used the Trentool Graph Correction function described in the manual (see http://www.trentool.de/ for more details).

In section Results, we will always report the *difference* between TE estimated on simulated signals, and that obtained from surrogate data. Whenever no statistical significance was achieved (*p* > 0.05) the difference was set at zero (i.e., no connection detected). Otherwise (connection detected), the true difference is used as an estimate of connectivity strength.

All details on the version of Trentool used and a table with all parameters adopted in the Trentool functions can be found in Supplementary Materials Part 1.

## Model Description

In the following, equations of a single region of interest (ROI) are described. Then, a model of several interconnected ROIs is built from these equations.

### Model of a Single Region of Interest

The model of a single Region of Interest (ROI) consists of the feedback arrangement among four neural populations: pyramidal neurons (subscript *p*), excitatory interneurons (subscript *e*), inhibitory interneurons with slow and fast synaptic kinetics (GABA_A, slow_ and GABA_A, fast_, subscripts *s* and *f* , respectively). Each population receives an average postsynaptic membrane potential (say *v*) from other neural populations, and converts this membrane potential into an average density of spikes fired by the neurons (say *z*). This conversion is simulated with a static sigmoidal relationship, which reproduces the non-linearity in neuron behavior (the presence of a zone where neurons are silent (below threshold) and an upper saturation, where neurons fire at their maximal activity).

**Figure 1:**
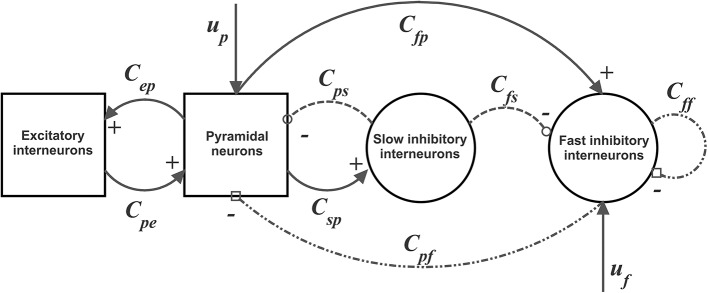
Block diagram of the neural mass model used to simulate the dynamics in a single ROI. Continuous lines with arrows denote glutamatergic excitatory synapses, dotted lines with open circles denote GABAergic slower inhibitory synapses, while dash-dotted lines with open squares denote GABAergic faster inhibitory synapses. Symbols *C*_*ij*_ represent the synaptic contacts among the neural populations where the first subscript denotes the post-synaptic population and the second subscript the pre-synaptic population. *u*_*p*_ and *u*_*f*_ represent the exogenous inputs to the ROI (from the external environment or form other ROIs), targeting the pyramidal and the fast inhibitory population, respectively.

To model dynamics in a whole ROI, the four populations are connected via excitatory and inhibitory synapses, according to the schema in Figure 1. Each synaptic kinetics is described with a second order system, but with different parameter values. We assumed three types of synapses: glutamatergic *excitatory* synapses with impulse response *h*_*e*_*(t)*, assuming that synapses from pyramidal neurons and from excitatory interneurons have similar dynamics; GABAergic inhibitory synapses with *slow* dynamics [impulse response *h*_*s*_*(t)*]; GABAergic inhibitory synapses with *faster* dynamics [impulse response *h*_*f*_*(t)*]. They are characterized by a gain (*G*_*e*_, *G*_*s*_, and *G*_*f*_, respectively) and a time constant (the reciprocal of these time constants denoted as ω_*e*_, ω_*s*_, and ω_*f*_,, respectively). The average numbers of synaptic contacts among neural populations are represented by eight parameters, *C*_*ij*_, where the first subscript represents the target (post-synaptic) population and the second refers to the pre-synaptic population.

In a previous work (Ursino et al., 2010) we performed a sensitivity analysis on the role of connections linking different ROIs, and found that the most influential connections are “from pyramidal to pyramidal” and “from pyramidal to fast inhibitory.” Accordingly, in this work we assume that inputs to each ROI (say *u*) target only pyramidal and fast-inhibitory populations (see Figure 1). The equations of a single ROI are written below:

Pyramidal neurons

(1) $$\begin{array}{l} \frac{dy_{p}\left(t\right)}{dt}=x_{p}\left(t\right) \end{array}$$

(2) $$\begin{array}{l} \frac{dx_{p}\left(t\right)}{dt}=G_{e}\omega_{e}z_{p}\left(t\right)\;-2\omega_{e}x_{p}\left(t\right)-\omega_{e}^{2}y_{p}\left(t\right) \end{array}$$

(3) $$\begin{array}{l} z_{p}\left(t\right)=\frac{2e_{0}}{1+e^{-rv_{p}}}-e_{0} \end{array}$$

(4) $$\begin{array}{l} v_{p}(t)=C_{pe}y_{e}\left(t\right)-C_{ps}y_{s}\left(t\right)\;-C_{pf}y_{f}\left(t\right) \end{array}$$

Excitatory interneurons

(5) $$\begin{array}{l} \frac{dy_{e}\left(t\right)}{dt}=x_{e}\left(t\right) \end{array}$$

(6) $$\begin{array}{l} \frac{dx_{e}\left(t\right)}{dt}=G_{e}\omega_{e}\left(z_{e}\left(t\right)+\frac{u_{p}\left(t\right)}{C_{pe}}\right)\;-2\omega_{e}x_{e}\left(t\right)-\omega_{e}^{2}y_{e}\left(t\right) \end{array}$$

(7) $$\begin{array}{l} z_{e}\left(t\right)=\frac{2e_{0}}{1+e^{-rv_{e}}}-e_{0} \end{array}$$

(8) $$\begin{array}{l} v_{e}\left(t\right)=C_{ep}y_{p}\left(t\right) \end{array}$$

Slow inhibitory interneurons

(9) $$\begin{array}{l} \frac{dy_{s}\left(t\right)}{dt}=x_{s}\left(t\right) \end{array}$$

(10) $$\begin{array}{l} \frac{dx_{s}\left(t\right)}{dt}=G_{s}\omega_{s}z_{s}\left(t\right)\;-2\omega_{s}x_{s}\left(t\right)-\omega_{s}^{2}y_{s}\left(t\right) \end{array}$$

(11) $$\begin{array}{l} z_{s}\left(t\right)=\frac{2e_{0}}{1+e^{-rv_{s}}}-e_{0} \end{array}$$

(12) $$\begin{array}{l} v_{s}\left(t\right)=C_{sp}y_{p}\left(t\right) \end{array}$$

Fast inhibitory interneurons

(13) $$\begin{array}{l} \frac{dy_{f}\left(t\right)}{dt}=x_{f}\left(t\right) \end{array}$$

(14) $$\begin{array}{l} \frac{dx_{f}\left(t\right)}{dt}=G_{f}\omega_{f}z_{f}\left(t\right)\;-2\omega_{f}x_{f}\left(t\right)-\omega_{f}^{2}y_{f}\left(t\right) \end{array}$$

(15) $$\begin{array}{l} \frac{dy_{l}\left(t\right)}{dt}=x_{l}\left(t\right) \end{array}$$

(16) $$\begin{array}{l} \frac{dx_{l}\left(t\right)}{dt}=G_{e}\omega_{e}u_{f}\left(t\right)\;-2\omega_{e}x_{l}\left(t\right)-\omega_{e}^{2}y_{l}\left(t\right) \end{array}$$

(17) $$\begin{array}{l} z_{f}\left(t\right)=\frac{2e_{0}}{1+e^{-rv_{f}}}-e_{0} \end{array}$$

(18) $$\begin{array}{l} v_{f}(t)=C_{fp}y_{p}\left(t\right)-C_{fs}y_{s}\left(t\right)\;-C_{ff}y_{f}\left(t\right)+y_{l}\left(t\right) \end{array}$$

The inputs to the model, *u*_*p*_*(t)* and *u*_*f*_*(t)* [Equations (6), (16)] represent all exogenous contributions coming from external sources (either from the environment or from other brain regions) filtered through the low-pass dynamics of the excitatory synapses [Equations (5), (6), (15), (16), respectively]. In fact, a common assumption in neurophysiology is that long-range connections in the brain are always mediated via excitatory glutamatergic synapses. In particular, *u*_*p*_*(t)* is the input to pyramidal cells and *u*_*f*_*(t)* the input to GABA_A, fast_ interneurons. These terms will be described below.

### Model of Several Interconnected ROIs and Connectivity Parameters

In order to study connectivity between regions, let us consider two ROIs [each described via Equations (1)–(18)], which are interconnected through long-range excitatory connections. The presynaptic and postsynaptic regions will be denoted with the superscript *k* and *h*, respectively. The generalization to more than two regions is trivial. Throughout the manuscript, we will use the first superscript to denote the target ROI (post-synaptic) and the second superscript to denote the donor ROI (pre-synaptic).

To simulate connectivity, we assumed that the average spike density of pyramidal neurons of the presynaptic area ($z_{p}^{k}$) affects the target region via a weight factor, $W_{j}^{hk}$ (where *j* = *p* or *f* , depending on whether the synapse targets to pyramidal neurons or fast inhibitory interneurons) and a time delay, *T*. This is achieved by modifying the input quantities $u_{p}^{h}$ and/or $u_{f}^{h}$of the target region.

Hence, we can write

(19) $$\begin{array}{l} u_{j}^{h}\left(t\right)=n_{j}^{h}\left(t\right)+W_{j}^{hk}z_{p}^{k}\left(t-T\right)\;j=p,f \end{array}$$

*n*_*j*_(*t*) represents a Gaussian white noise (in the present work, if not explicitly modified, we used: mean value *m*_*j*_ = 0 and variance σ_*j*_^2^ = 9/*dt*, where *dt* is the integration step) which accounts for all other external inputs not included in the model.

It is worth noting that the synapses $W_{p}^{hk}$ have an excitatory role on the target region *h*, since they directly excite pyramidal neurons. Conversely, synapses $W_{f}^{hk}$, although glutamatergic in type, have an inhibitory role, via a bi-synaptic connection. In particular, both connections go from the source ROI *k* to the target ROI *h*, but in the inhibitory case this is composed of two synapses (from pyramidal neurons in the source ROI *k* to inhibitory interneurons in the target ROI *h* and then from inhibitory interneurons in target ROI *h* to pyramidal neurons still in ROI *h*). Hence, the second synapse is internal to ROI *h* and has not been modified throughout this work. Hence, in the following the general terms “excitatory connection” and “inhibitory bi-synaptic connection” will be used to describe these two different connections, although both glutamatergic in type. In particular, we wish to stress that the Dale principle is always satisfied in our model, since individual neural populations within each ROI are either excitatory or inhibitory, and this distinction is established a priori in the model.

In line with the notation used for the inter-region synapses, in the following we will denote with *TE*^*hk*^ the transfer entropy from ROI *k* to ROI *h*, that is *TE*^*hk*^ = *TE*(*ROI k* → *ROI h* ).

### Assignment of Model Parameters

**Figure 2:**
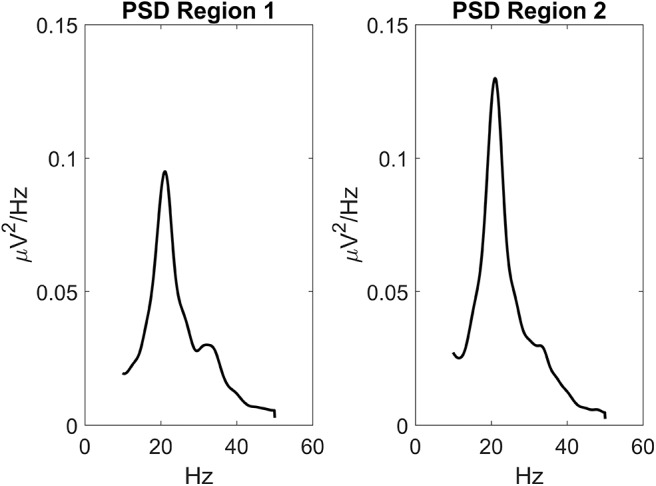
Power spectral density simulated with the model assuming two regions interconnected via excitatory synapses ($W_{P}^{12}=40$ and $W_{P}^{21}=60$) and no inhibitory connections ($W_{F}^{12}=0$ and $W_{F}^{21}=0$). Parameters within the neural mass models are reported in Table 1, and maintained for all simulations in this work. The input to the two regions was a random white noise with zero mean values and variance 9/dt (where dt is the integration step, hence the power density of random noise is 9). In this conditions, the two regions exhibit a clear oscillation in the beta range (about 20 Hz) with a contribution in the gamma range too (about 35 Hz). This pattern is similar to the one observed in premotor and supplementary motor areas (Zavaglia et al., 2008, 2010; Ursino et al., 2010). It is worth noting that the second region exhibits greater power, since it receives higher excitation.

Parameters within each ROI were given to simulate a power spectral density with a significant activity in the beta range (about 20 Hz) and some activity in the gamma range (above 30 Hz see Figure 2). This power density is typical of supplementary and pre-motor cortical areas (see also Zavaglia et al., 2008; Ursino et al., 2010; Cona et al., 2011). Power spectral density was computed by applying the Welch method on the post-synaptic membrane potential of pyramidal neurons (i.e., on quantity *v*_*p*_ in Equation (4), which is representative of local mean field potentials). Figure 2 was obtained assuming that the two ROIs are linked with excitatory connections. Of course, power density can change if other kinds of synaptic connections among ROIs are implemented, still keeping these two main rhythms as they depend on the internal parameters of each ROI.

**Table 1:** Parameters setting used in the Neural Mass Model to simulate dynamics in a single ROI.

**Internal parameters**	
**Connectivity constants**	
*C*_*ep*_	40
*C*_*pe*_	40
*C*_*sp*_	40
*C*_*ps*_	50
*C*_*fs*_	20
*C*_*fp*_	40
*C*_*pf*_	60
*C*_*ff*_	20
**Reciprocal of synaptic time constants:**	
ω_*e*_	75 *s*^−1^
ω_*s*_	30 *s*^−1^
ω_*f*_	300 *s*^−1^
**Synaptic gains:**	
*G*_*e*_	5.17 *mV*
*G*_*s*_	4.45 *mV*
*G*_*f*_	57.1 *mV*
**Saturation value of the sigmoid:**	
*e*_0_	2.5 *Hz*
**Slope of the sigmoid:**	
*r*	0.56 *mV*^−1^

A list of parameters for the average numbers of intra-region synaptic contacts *C*_*ij*_, and for the reciprocal of synaptic time constants, ω_i_, is reported in Table 1. These internal parameters have been maintained constant and equal for all ROIs throughout the following simulations.

As stated previously, for each model configuration 10 simulations were repeated and the model output signals (post-synaptic membrane potentials *v*_*p*_ of each ROI) of these 10 trials fed as input to software Trentool, for TE estimation and comparison with surrogate data. The length of simulated signals was 60 s in general, but the effect of signal length on TE estimation was also assessed (see section Results). Finally, it is important to remark that for each model configuration, the simulations were performed using always the *same 10 seeds* to realize white noise; hence TE differences among model configurations can be ascribed only to differences in synapses (or differences in the input mean value or variance), not to individual random noise realizations.

In order to gain a deeper understanding of the virtues and limitations of TE, in all cases the results of TE estimates were compared with those obtained with a linear delayed correlation coefficient (DCC). For the sake of brevity, all results of the DCC are reported in Supplementary Material Part 2.

## Results

### Two Interconnected ROIs

A first set of simulations was performed by using two ROIs, linked by means of reciprocal inhibitory and/or excitatory connections.

**Figure 3:**
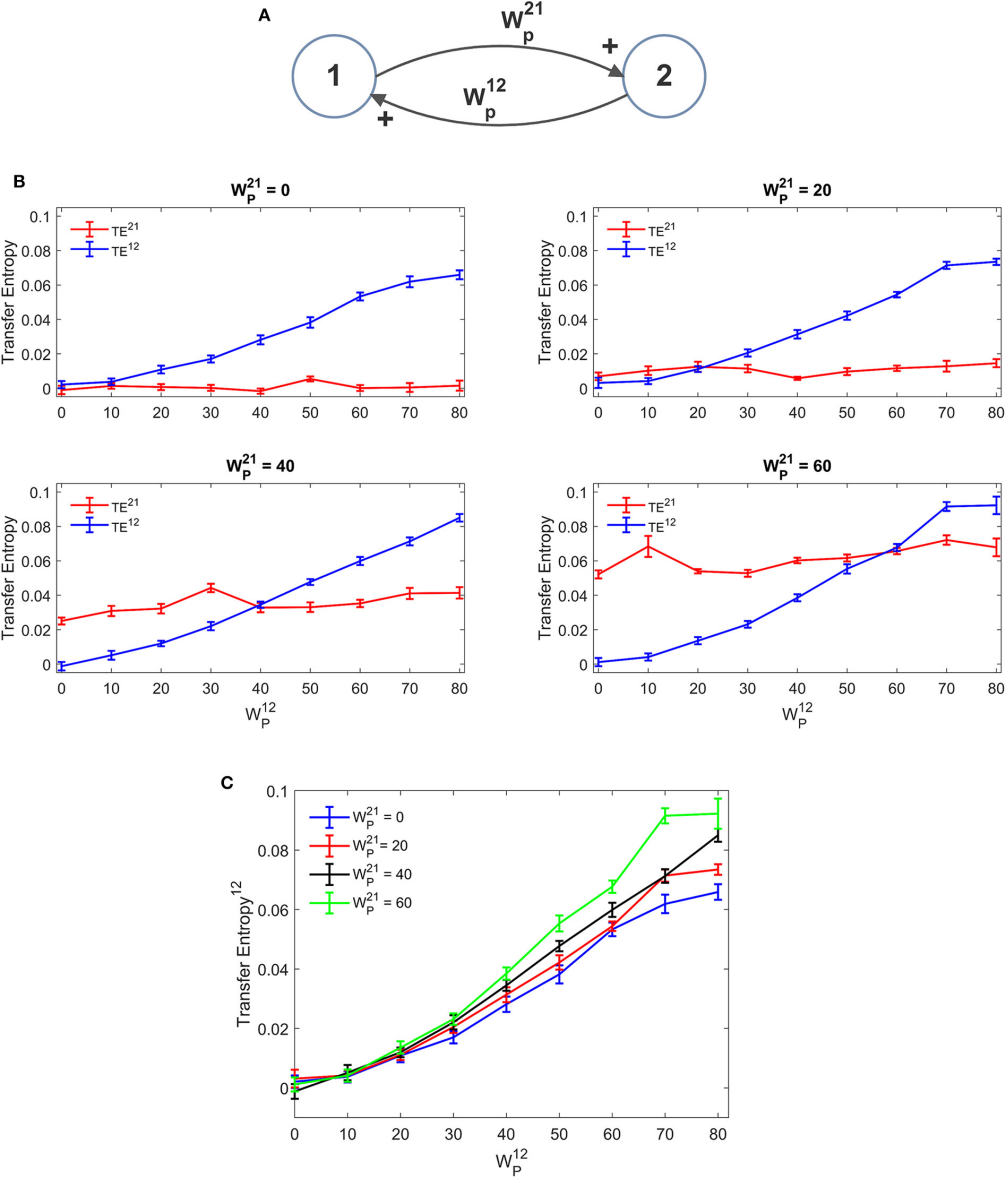
Dependence of Transfer Entropy on a feedback, realized assuming two regions interconnected with reciprocal excitatory synapses **(A)**. In particular the synapse from region 2 to region 1 ($W_{P}^{12}$) was progressively varied between 0 and 80, at different values of the synapse from region 1 to region 2 ($W_{P}^{21}$). Inhibitory synapses were set at zero. **(B)** Reports the individual values [TE^12^ and TE^21^ ± Standard Error of the Mean (SEM)], obtained with all combinations of synapses. Results concerning the transfer entropy TE^12^ from region 2 to region 1 are further summarized in **(C)**. As it is clear, TE increases quite linearly with the value of the excitatory synapse in the direction under study, and it is also moderately affected by the value of the excitatory synapse in the other direction.

Figure 3 depicts the TE estimated when the two ROIs are linked via two *excitatory* connections, realized by means of pyramidal-pyramidal synapses $W_{P}^{12}$ and $W_{P}^{21}$, in the absence of any reciprocal inhibitory link. Some aspects of the results are noticeable: (i) when the synapse is zero, the relative TE is negligible (i.e., not significantly different from that of surrogate data); (ii) TE increases quite linearly with the strength of the synapse; (iii) TE from region 2 to 1 increases moderately when the reciprocal synapse (i.e., $W_{P}^{21}$ from ROI1 to ROI2) increases. This effect is made evident by the greater slope in the linear relationships of Figure 3C. By comparing the results of the TE with those obtained with the DCC (see Figure S3) one can observe that synapse strength estimation with TE is more reliable and less affected by the changes in the other synapse; DCC can discriminate between the two synapse strengths (i.e., it is a bidirectional estimator) but estimation of one synapse tends to increase more markedly with the increase in the other.

**Figure 4:**
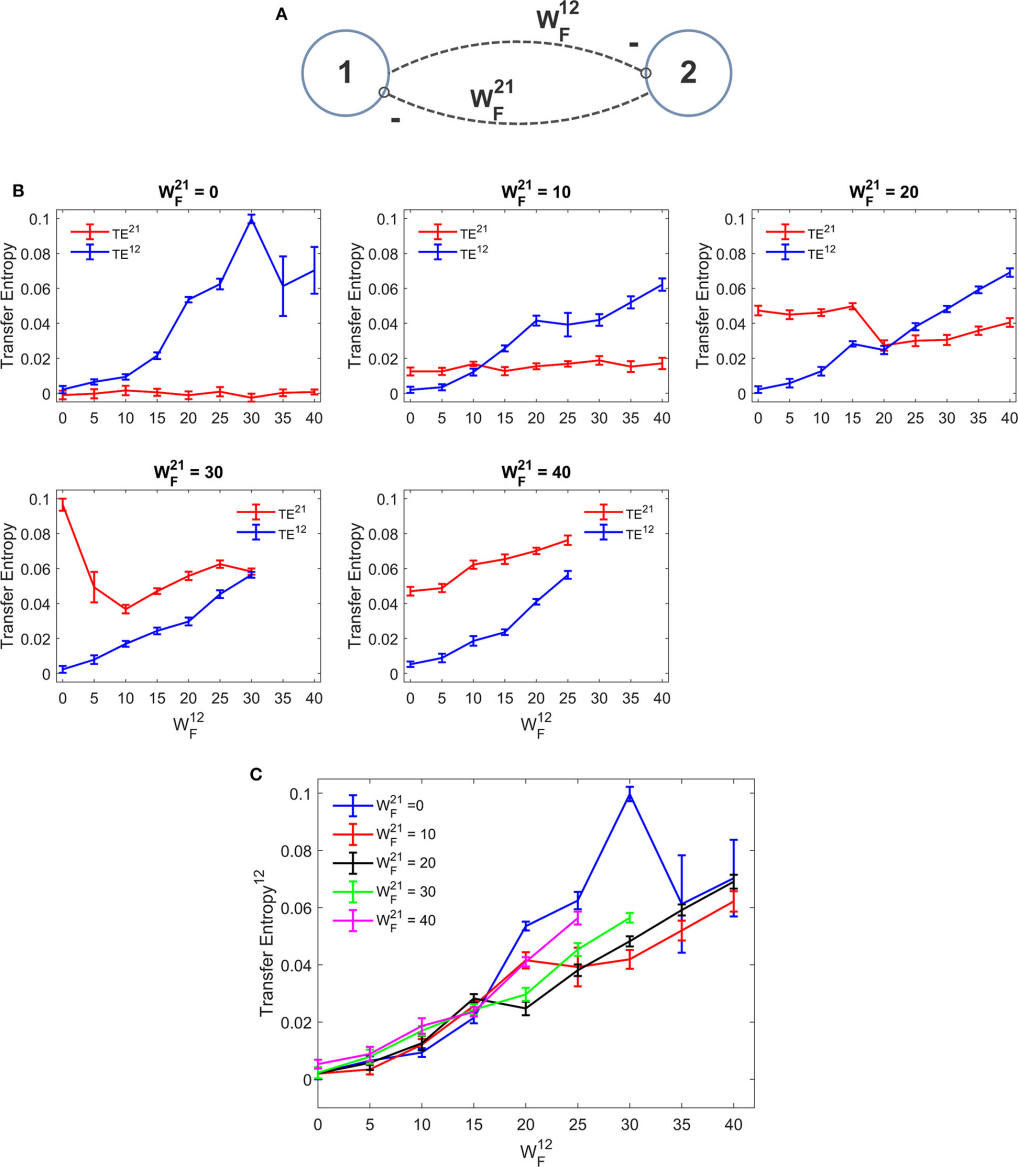
Dependence of Transfer Entropy on a feedback realized assuming two regions interconnected with reciprocal inhibitory synapses **(A)**. In particular the synapse from region 2 to region 1 ($W_{F}^{12}$) was progressively varied between 0 and 40, at different values of the synapse from region 1 to region 2 ($W_{F}^{21}$). If both inhibitory synapses are too high, the algorithm fails to compute acceptable values of TE. Excitatory synapses were set at zero. **(B)** Reports the individual values (TE^12^ and TE^21^ ± SEM), obtained with all combinations of synapses. The results concerning the transfer entropy TE^12^ from region 2 to region 1 are further summarized in **(C)**. As it is clear, TE increases with the value of the inhibitory synapse in the direction under study; but the value of the inhibitory synapse in the other direction affects the estimation significantly. It is worth noting that the effect of inhibitory synapses on TE is stronger than the effect of excitatory synapses [let us compare results of **(B,C)** with those in **(B,C)**].

Figure 4 depicts the TE estimated when the two ROIs are linked via reciprocal bi-sinaptic *inhibitory* connections. Results substantially confirm that TE increases quite linearly with the synapse strength. However, some differences are evident compared with the excitatory case. First, the effect of an inhibitory connection on TE is much more efficacious than the effect of an excitatory link. In fact, an increase in the synapse $W_{F}^{12}$ from 0 to 25 to 30 causes an increase in TE from 0 to ~0.06 in our simulated data. To produce the same effect, an excitatory synapse (say $W_{P}^{12}$) should be increased from zero to ~60. Hence, in our particular model realization, inhibitory connections are about 2-fold more efficacious in information transmission compared with the excitatory connections. Second, we observed a peak in the estimation of TE when one synapse (either $W_{F}^{12}$ or $W_{F}^{21}$) is set at zero and the other has a value as high as 30. Assuming that this peak represents a failure in the algorithm accuracy, we repeated the estimations of TE using a greater number of trials (30 instead of 10). We observed that, with 30 trials the peak in Figure 4C disappears (i.e., we have a TE value as low as 0.0723 for $W_{F}^{12}$ = 30 and $W_{F}^{21}$ = 0, while the other values remain very similar to those computed with 10 trials).

In some other cases (when $W_{F}^{21}$ = 30 or 40 and $W_{F}^{12}$ greater than 25) Trentool fails to find a correct solution; the problem here is related with the reconstruction of states from scalar time series using time-delay embedding. In particular, TRENTOOL tries to optimize both the embedding dimension and the embedding delay according to Ragwitz' criterion (see Trentool manual); this procedure provides an error in these particular cases.

Comparison with DCC (Figure S4) shows that correlation can be used to detect the sign of the synapse (i.e., DCC provides negative value in case of inhibitory connections, whereas TE is always positive) and is more regular (i.e., it does not exhibit sudden peaks). However, in this case too, as in Figure S3, the synapse strength estimation by DCC increases markedly with an increase in the reciprocal synapse.

### Three Connected ROIs

Various simulations were performed by assuming three interconnected ROIs (named 1, 2, and 3 in the following) with reciprocal connections (either inhibitory or excitatory). This is a multivariate condition; for instance, the estimated TE^12^ from ROI2 to ROI1 may be affected also by the connections between ROI1 and ROI3 and between ROI2 and ROI3. Hence, we expect that results are much less linear than in the previous case.

**Figure 5:**
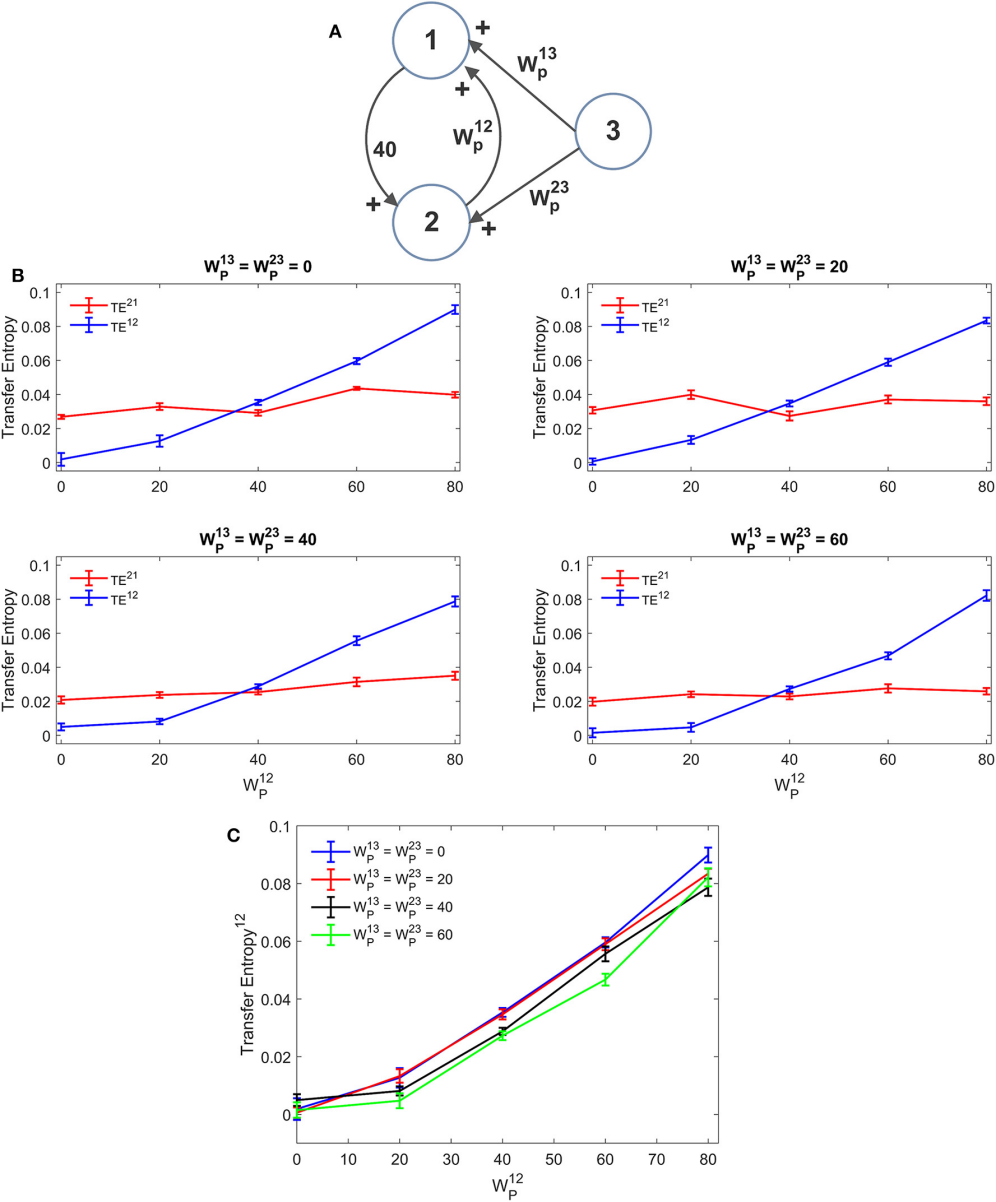
Influence of a common external source on TE estimation. Simulations were performed assuming three regions (see **A**) interconnected via an excitatory synapse from region 2 to region 1 ($W_{P}^{12}$), which was progressively varied between 0 and 80, and a constant excitatory synapse in the other direction set at the value $W_{P}^{21}=40$. The two regions 1 and 2 also receive a shared input coming from the third region, via equal excitatory synapses $W_{P}^{13}=W_{P}^{23}$. **(B)** reports the individual values of TE (TE^12^ and TE^21^ ± SEM), obtained at different strength of the input from region 3. The results concerning the transfer entropy TE^12^ from region 2 to region 1 are further summarized in **(C)**. TE increases linearly with the value of the excitatory synapse and is quite independent of the presence of an external shared input.

A first simulation was performed assuming that ROI1 and ROI2 receive a common input from a third region (ROI3). The schema is depicted in Figure 5A. The strength of this common input was then progressively raised (Figures 5B,C). Results suggest that estimation of TE is only moderately affected by the presence of a shared input. An increase of this input causes just a moderate reduction in TE, which endangers linearity especially at low values of the synapse $W_{P}^{12}$. A similar independence on the shared input can be observed looking at the DCC, too (Figure S5). However, it is worth noting that, in these simulations, we used the same delays (16.5 ms) for all connections: this could induce a resonance. More complex conditions, using different delays, can be tested in future studies.

**Figure 6:**
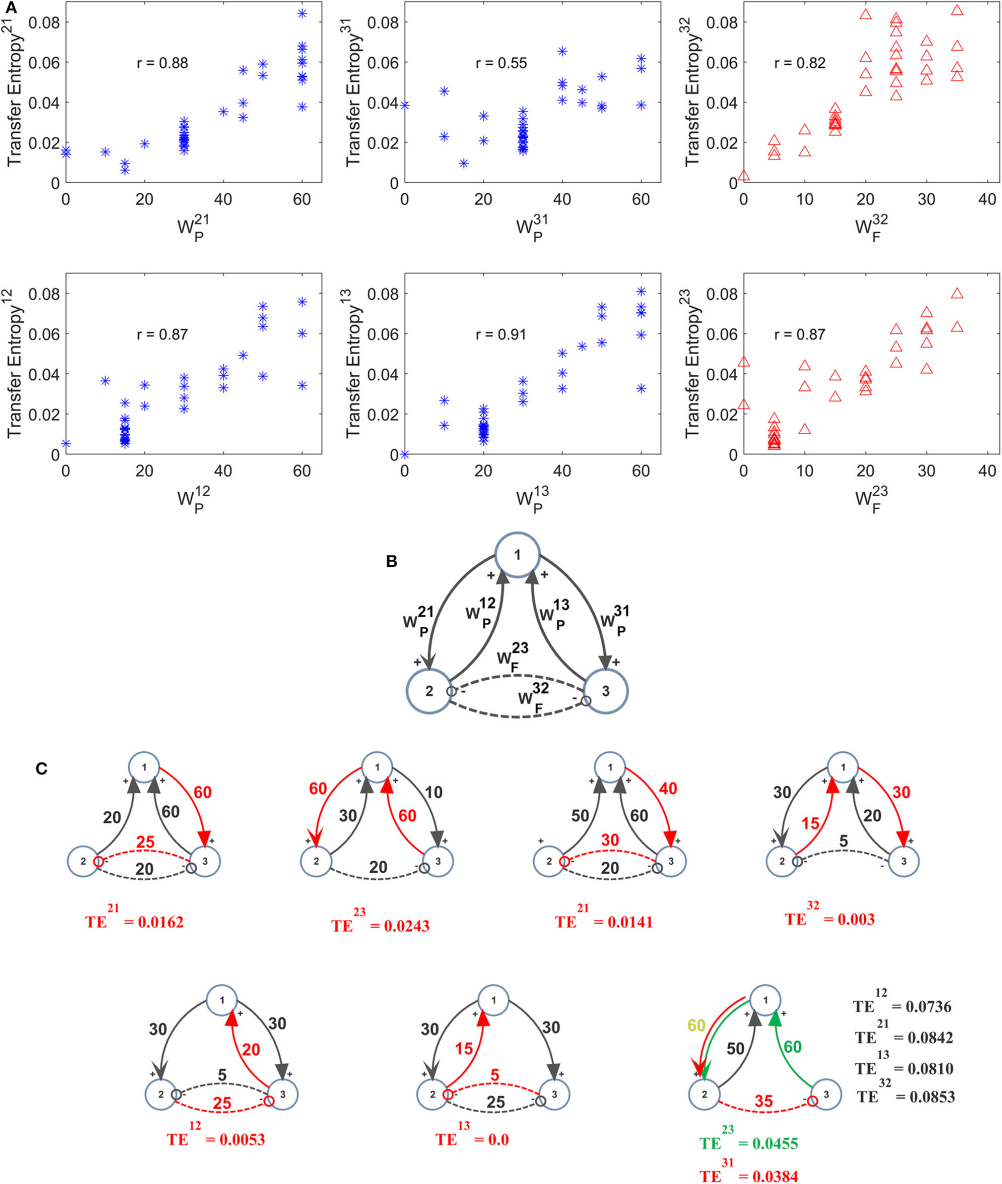
Effect of different combinations of synapses on TE in a model of three interconnected regions. Simulations were performed by using the schema depicted in **(B)**, where regions 2 and 3 are in competition via inhibitory synapses ($W_{F}^{32}\;$and$W_{F}^{23}\;$), and are linked via excitatory synapses to region 1 (synapses $W_{P}^{12}\;$, $W_{P}^{21}\;$, $W_{P}^{13}\;$, and $W_{P}^{31}\;$). All other synapses are set at zero. Thirty-eight simulations were performed with various combinations of the six synapses described above. The panels in **(A)** show the TE in a given direction, as a function of the synapse in the same direction, while the other synapses were varied (SEM are not reported here for simplicity, but are of the same order as in the other figures). Quite a high positive correlation is evident; however, the effect of the other synapses has a strong role in modulating the value of TE. The snapshots in **(C)** summarize all simulations performed with at least one synapse set at zero. A spurious TE can be ascribed to the presence of a bi-synaptic link (red or green lines). Only in the last snapshots (right bottom), all values of TE are reported, to compare the spurious values of TE with those of real synaptic links.

Further three-ROIs simulations were performed using the more complex schema depicted in Figure 6B, where ROI2 and ROI3 are in competition via reciprocal inhibitory synapses, and exchange excitation with ROI1. Thirty-eight different combinations of excitations and inhibitions were tried. Since results are quite numerous, we do not describe all cases in detail, but just a global summary is reported in the plots of Figure 6A. In these plots we show the value of TE estimated in a single pathway, as a function of the synapse strength used in that path, while the others synapses are varied (for instance, in the upper left panel in Figure 6A, $W_{P}^{21}$ is varied from 0 to 60, while the other synapses are varied, for a total of 38 different simulations). As it is clear from this figure, despite the multivariate condition, quite a linear relationship is maintained between the estimated value of TE and the synapse value in that pathway; however, the correlation between the two quantities decreases significantly compared with the univariate case.

In seven cases out of 38 in Figure 6, at least one synapse was set at zero. However, due to the presence of a multivariate condition, a residual TE is computed by the algorithm despite the absence of a direct causal link. The situation is summarized in the seven snapshots of Figure 6C, where the “spurious” TE value (i.e., the value associated with the null synaptic connection) is reported, together with the network generating such a false estimate. It is worth noting that the spurious TE is always the consequence of a bi-synaptic link (highlighted in red), and its value quite regularly reflects the strength of this link. However, the spurious TE is always quite small (<0.025). Only in the last snapshot, where two synapses are simultaneously set at zero, spurious TE values increase to ~0.04 or more. However, they are still much smaller than TE values associated with “true” synapses.

By comparing the results in Figure 6 with those obtained with the DCC (Figure S6) one can observe a similar behavior in the estimation of most synapses. The main difference is that TE provides a much better estimation of the inhibitory synapse ${\text{W}}_{\text{F}}^{32}$ than DCC. DCC, however, is able to discriminate between excitatory connections and inhibitory bi-synaptic connections, providing negative values in the last case.

### Four Connected ROIs

**Figure 7:**
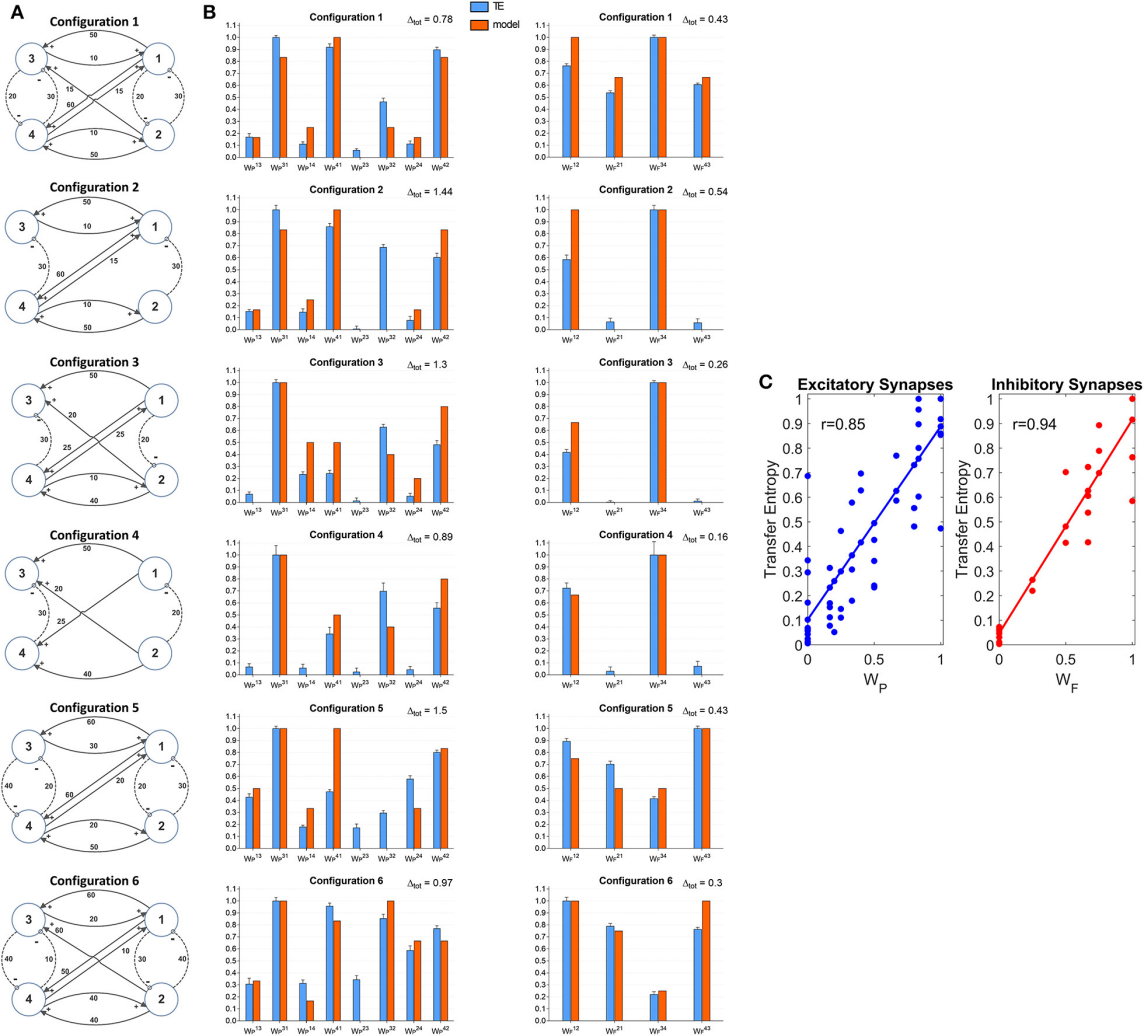
Estimation of the connectivity strength obtained during six different simulations, each performed with four interconnected ROIs. Each row refers to a different network configuration (see reported in **A**) in which the ROIs 1 and 3 belong to one hemisphere, and the ROIs 2 and 4 to another hemisphere. Each ROI exchange *inhibitory* connections with the adjacent ROI at the same layer in the other hemisphere (1 vs. 2 and 3 vs. 4), and feedback excitatory connections with ROIs of the other layer (1 and 2 vs. 3 and 4). Bars in the two columns of **(B)** compare the estimated TE values (±SEM) with the true connectivity values in each circuit, normalized to the maximum (the graph bar in the first column considers the eight excitatory synapses, the graph bar in the second column the four inhibitory synapses). Finally, **(C)** reports the correlation between all the estimated TE values and the true connectivity values, normalized to the maximum, for the excitatory and the inhibitory synapses.

A further set of simulations was performed using four interconnected ROIs. In order to mimic a physiological schema, we assumed that two ROIs represent regions located in the left hemisphere (ROIs 1 and 3) and the other two represent regions in the right hemisphere (ROIs 2 and 4). Moreover, we assumed that excitation in one cortex can lead to inhibition of the symmetrical area in the other cortex and vice versa, according to the Theory of Inhibition (see Mangia et al., 2017), whereas feedback excitations can be present between the previous layer and the subsequent layer. A similar schema may occur, for instance, considering the connections between the two Supplementary Motor and the two Primary Motor areas (Grefkes et al., 2008; Pool et al., 2018). Six different networks, which differ as to the number and strength of connections were simulated (Figure 7A). A comparison between the estimated TE values and the model synaptic strengths is reported in Figure 7B (in all cases, to allow a direct comparison, the values are normalized to the maximum for each configuration). As it is clear from this figure, just in a few cases TE can produce some spurious connections (*one* ${\text{W}}_{\text{P}}^{32}$ in the configuration n. 2, two ${\text{W}}_{\text{P}}^{23}$ and ${\text{W}}_{\text{P}}^{32}$ in the configuration n. 5 and one $W_{P}^{23}$ in the configuration n. 6, if we consider a threshold as low as 0.1 to discriminate between the presence or the absence of a synapse). In most cases, TE overestimates the synapse $W_{P}^{32}$. Synapses $W_{P}^{14}$ and ${\text{W}}_{\text{P}}^{41}$ are underestimated in the configurations 3 and (especially $W_{P}^{41}$) in the configuration 5. In general, however, the overall behavior is satisfactory, with a high correlation between the normalized synaptic strengths of the models and the normalized TE values (Figure 7C). It is worth-noting that estimation of the inhibitory by-synaptic connections is more reliable than the estimation of the excitatory connections.

A comparison with Figure S7 shows that TE is much more reliable compared with the DCC in the evaluation of 4 interconnected ROIs. Briefly, the number of spurious connections is higher, the difference between the normalized DCC values vs. the normalized model synaptic values is higher, and the correlation between the DCC values and the true synaptic weights much poorer when using DCC than TE.

### Effect of the Input Mean Value and SD

The neural mass model used in this work is intrinsically non-linear (due to the presence of sigmoidal relationships which mimic the dependence of spike density on post-synaptic membrane potential in individual neural populations). Conversely, most methods used to assess connectivity from data (like PDC or most implementations of DCM) assume a linear model in the estimation process. TE does not assume any model behind signals, but simply computes information transfer from the source to the target.

**Figure 8:**
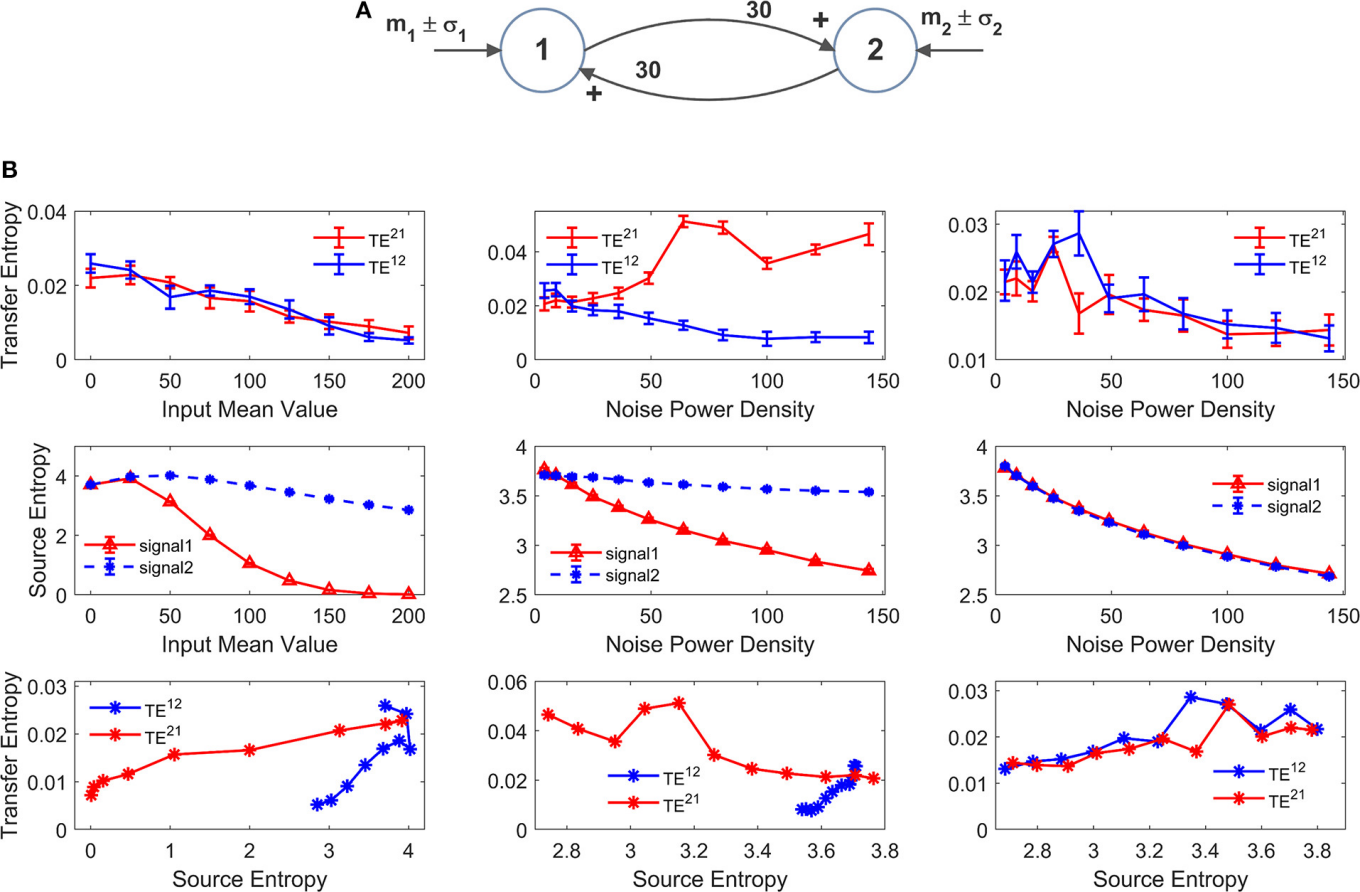
Effect of the mean value and standard deviation of the input noise on the estimation of Transfer Entropy. The simulations were performed using two interconnected regions **(A)**, with synapses $W_{P}^{12}\;=W_{P}^{21}=\text{30}$, $W_{F}^{12}\;=W_{F}^{21}=\text{0}$ and by varying the mean value m_1_ (left panels in **B**) and standard deviation σ_1_ of noise (middle panels in **B**) of the input to ROI1. Standard deviation (σ) of the noise was computed as σ =$\sqrt{\rho /dt}$ where *dt* is the integration step and ρ is the noise power density. Finally, the right panels in **(B)** show the case when noise standard deviation was increased in both populations altogether (both parameters σ_1_ and σ_2_). In **(B)**, the first row shows TE ± SEM, while the second row shows entropy (±SEM) of the two signals, vs. the input values. Finally, the third row in **(B)** plots the TE vs. the entropy of the source signal. It is worth noting the presence of quite a linear dependence of TE on the source entropy, with the only significant exception of TE^21^ in the middle panel.

**Figure 9:**
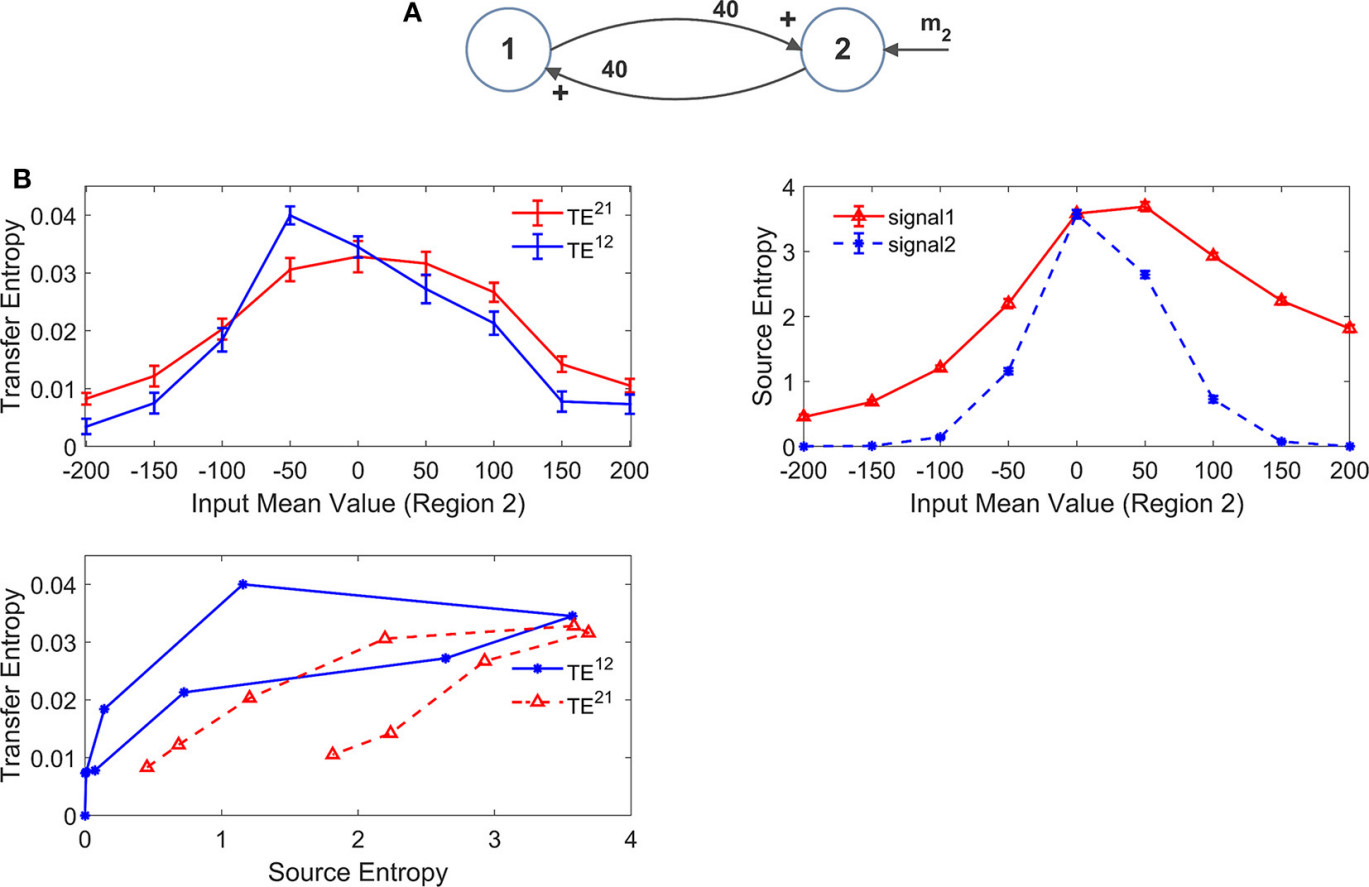
Effect of the region working point on the estimation of Transfer Entropy. The simulations were performed using two interconnected regions **(A)**, with synapses $W_{P}^{12}\;=\text{40}$, $W_{P}^{21}\;=4\text{0}$, $W_{F}^{12}\;=\text{0}$, and $W_{F}^{21}\;=\text{0}$ (i.e., just a reciprocal excitation). In this case we assumed that input to pyramidal neurons in region 1 have a zero mean value, whereas mean value m_2_ of the input to pyramidal neurons of region 2 is progressively increased from −200 (strong inhibition) to +200 (strong excitation). In **(B)**, the left plot shows TE ± SEM, while the right top plot shows entropy (±SEM) of the two signals, vs. the input values. Finally, the bottom left plot show the TE vs. the entropy of the source signal. As it is clear, transfer entropy reaches a maximum value when the first region exits from the inhibition zone to the central zone. Furthermore, TE declines when regions enter into the upper saturation, due to excessive excitation. It is worth noting the presence of quite a linear dependence of TE on the source entropy, although with a hysteresis.

However, it is to be stressed that, due to non-linear effects, information transfer may not reflect true anatomical connectivity. Hence, it is of the greatest value to assess how TE estimation may change in conditions when all synapses are fixed (i.e., a constant anatomical connectivity is used) but the working point or the global activity in the neural populations is modified. Results of this analysis are reported in Figures 8, 9 as detailed below. For the sake of simplicity, we show results obtained using two-ROIs model, with the two ROIs connected via reciprocal excitatory synapses (schemes in Figures 8A,9A).

In order to test non-linear phenomena, first we modified either the mean value or the standard deviation of the noise entering to pyramidal neurons in one ROI. Indeed, a change in the mean value shifts the working point along the sigmoidal relationship. An increase in SD causes large oscillations in neuronal activity, which may be partly cut-off by the saturation levels of the sigmoid. We also tested the effect of changing the standard deviation of the noise to both ROIs. We remark that all previous simulations were performed with zero mean values and variance σ^2^ = 9/*dt*, where *dt* is the integration step.

Since these changes may induce a change in the entropy of the source, we also computed the entropy of the two signals and we evaluated the relationship between TE and the entropy of the source signal.

The left panels in Figure 8B show the effect of an increase in the mean value of the input to ROI1 (parameter m_1_ in Figure 8A). Increasing this value causes a significant decline in the estimated value of the TE from 1 to 2. The reason is that activity of pyramidal neurons in ROI1 approaches the upper saturation, hence its entropy is dramatically reduced, and the quantity of information transmitted from 1 to 2 is reduced too. It is worth noting that also TE from 2 to 1 decreases. The reason is that also ROI2 exits from the central linear region, as a consequence of the strong excitation coming from ROI1, thus causing a moderate reduction in its entropy.

The middle panels in Figure 8B shows that an increase in the standard deviation of noise entering into ROI1 (parameter σ_1_ in Figure 8A, whereas noise to ROI2 is maintained at the basal level) causes a dramatic increase of TE from 1 to 2, despite a reduction in the entropy of signal 1. At the same time, TE from 2 to 1 is reduced. This result indicates that the values of TE do not only reflect the connectivity strength, but also the reciprocal level of noise in the two ROIs.

Finally, in the right panel of Figure 8B we tested the case when both standard deviations (to ROI1 and to ROI2, i.e., parameters σ_1_ and σ_2_ in Figure 8A) are progressively increased altogether. In this condition, both TEs show a similar decrease, but the changes are quite moderate compared with the previous cases, and reflect the moderate decrease in both source entropies.

Looking at the bottom row in Figure 8B, we can observe the presence of quite a linear relationship between TE and the entropy of the source. There is only one remarkable exception; the increase in noise of signal 1 in the middle panel reduces its entropy (due to a saturation) but causes an increase in information transmission from 1 to 2, which is reflected in a negative relationship between TE and the source entropy.

Similar results can be obtained using the DCC, as shown in Figure S8.

The effect of the input mean value is further illustrated in Figure 9B where we modified the input mean value entering to ROI2 (parameter m_2_ in Figure 9A). Here we assume that ROI2 starts from a condition of strong external inhibition (obtained with a negative input mean value). In this initial state, TE is almost zero despite the presence of a strong reciprocal connectivity. Then, the input to ROI2 is progressively increased (i.e., the pyramidal population is progressively excited). As a consequence of this excitation, ROI1 is excited too. In this situation, TE initially increases as a consequence of progressive reciprocal excitation in the network, which corresponds to an increase in the entropy of both signals. When excitation becomes excessive, however, TE starts to decrease (as in the example of Figure 8) since both regions enter into the upper saturation zone, and the source entropies decrease again. The relationship between TE and the entropy of the source (bottom left panel in Figure 9B) is quite linear, although it exhibits a kind of hysteresis.

In this case too, the patterns obtained with the DCC are similar (Figure S9).

### Estimation of the Pure Delay

In our model we included a pure delay in the connectivity among the different ROIs. This simulates the time necessary for spikes to travel along axons and reach a target region starting from a source region. During the previous simulations we used a pure delay as high as 16.5 ms in all synapses. The software package Trentool provides an estimation of this delay, assuming the value which maximizes TE.

In order to assess the role of pure delay, we repeated some simulations with two interconnected ROIs by varying the delay. We examined whether: (i) the value is correctly estimated by the algorithm (at least approximately); (ii) the estimated value of TE is affected by this delay.

**Figure 10:**
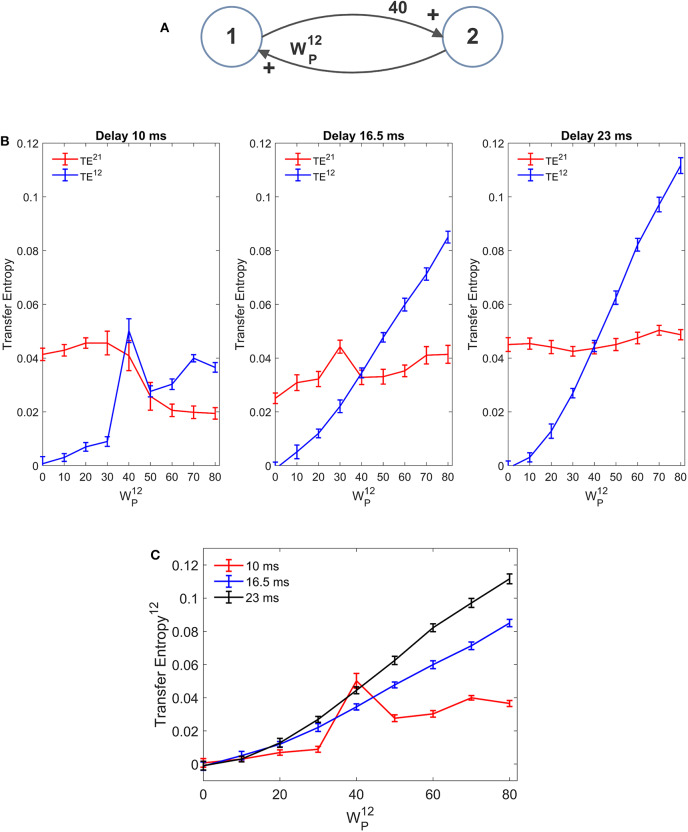
Effect of the delay between the two regions on the estimation of Transfer Entropy. The simulations were performed using two interconnected regions **(A)**, with synapses, $W_{P}^{21}\;=4\text{0}$, $W_{F}^{12}\;=\text{0}$, and $W_{F}^{21}\;=\text{0}$, and by changing the value of synapse $W_{P}^{12}$ (hence we have a reciprocal excitation). The simulations were repeated with different delays. It is worth noting that the delay value was estimated by the algorithm together with TE. **(B)** Reports the individual values (TE^12^ and TE^21^ ± SEM), obtained with all combinations of synapses. The results concerning the transfer entropy TE^12^ from region 2 to region 1 are further summarized in **(C)**. The estimation of TE increases (both as to its strength and linearity) at high values of time delay, and worsens when time delay is reduced.

Results, summarized in Figure 10 (with $W_{P}^{12}$ varying and $W_{P}^{21}$ fixed), show that the estimated value of TE is reduced, and the linearity in the relationship “TE vs. synapse strength” worsens if a small value is used for the delay (10 ms). Conversely, larger values (16.5 or 23 ms) provide robust results, with a moderate increase in TE with larger delays. The values of delay estimated by the algorithm are 8 ms, when we used a delay as low as 10, 15 ms when we use a delay as large as 16.5, and 25 ms when we use a delay as large as 23 ms.

In the left panel of Figure 10B we can observe an anomalous peak in TE when $W_{P}^{12}$ = 40. In this case too, as in the case of Figure 4C, this peak could be eliminated using 30 trials in the computation of TE (TE = 0.0199).

It is worth noting that 10 ms are about 1/5 of the resonant period of the present model (see the spectra in Figure 1), whereas 16.5 is about 1/3 of this period and 23 ms close to ½. This may have an impact in the synchronization of the two circuits.

Results obtained with the DCC (Figure S10) are similar. DCC appears more robust than TE when using a small delay, but even in this case the sensitivity of the estimator (i.e., the slope of the relationship between the metrics and the synapse strength $W_{P}^{12}$) increases with the delay. Moreover, as usual, DCC fatigues to assess a constant synapse ($W_{P}^{21}$ fixed) when the other synapse is varying, at all values of the delay.

### Effect of the Signal Length

**Figure 11:**
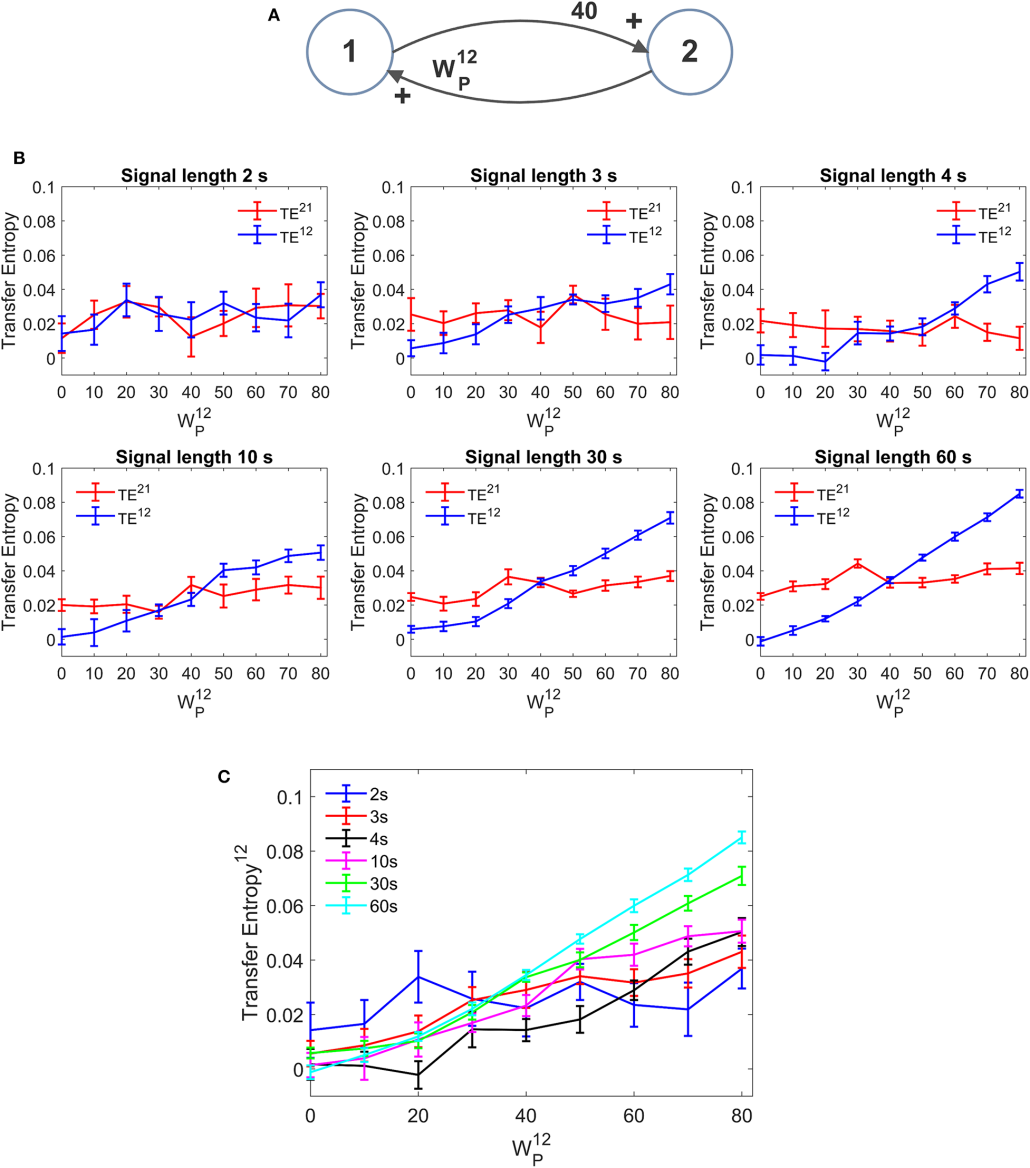
Effect of the duration of the signal on the estimation of Transfer Entropy. The simulations were performed using two interconnected regions **(A)**, with synapses, $W_{P}^{21}\;=4\text{0}$, $W_{F}^{12}\;=\text{0}$, and $W_{F}^{21}\;=\text{0}$, and by changing the value of synapse $W_{P}^{12}$ (hence we have a reciprocal excitation). The simulations were repeated with different durations of the signals. **(B)** Reports the individual values (TE^12^ and TE^21^ ± SEM), obtained with all combinations of synapses. The results concerning the transfer entropy TE^12^ from region 2 to region 1 are further summarized in **(C)**. The estimation of TE and its linear relationship with the synaptic strength are reduced when the signal length becomes as low as 3–4 s and, at shorter length, the algorithm totally fails to detect a linear relationship between TE and connectivity (let us note that the relationship between TE^12^ and synapse $W_{P}^{12}$ becomes flat when the signal length is as short as 2 s).

An important aspect in the estimation of TE is the signal length. In neural problems non-stationarity often precludes the use of long signals; the use of short signals, in turn, may jeopardize the estimation accuracy. In all previous simulations we used long stationary signals (60 s of simulations with fixed parameters and a stationary random noise, evaluated after settling the initial transient phenomena). We repeated simulations using a useful signal length (after elimination of the initial transient period) as low as 30, 10, 4, 3, and 2 s. It is worth noting that, even when using the shorter temporal window, at least 40 cycles of network oscillations are contained within the examined portion of the signal. Results are summarized in Figure 11. We can observe that, in the range 3–60 s, a reduction in signal length causes a reduction in the estimated value of TE, but the linearity in the relationship “TE vs. synapse strength” is approximately preserved even with the use of shorter signals (although linearity becomes more evident for signal length ≥ 10 s). Hence, caution must be taken when comparing TE values obtained from signals with different length. Conversely, when the signal is as short as 2 s, TE becomes quite insensitive to the synapse strength, although a value of TE significantly different from that of surrogate data is still detectable.

Conversely, DCC (Figure S11) is almost no affected by the signal length. Once again, however, DCC fails to assess that a synapse is constant when the other is varying, whereas TE can recognize a constant synapse even when using short signals.

## Discussion

In the last decade the use of methods to assess connectivity from neuroimaging data has received an enormous attention, as a fundamental aspect of cognitive neuroscience (Rossini et al., 2019). In fact, adequate understanding of brain functioning can be obtained only by considering the brain as a fully integrated system, the parts of which continuously exchange information in a dynamical reciprocal way (Sporns, 2011, 2013). However, much debate is still present in the literature on the reliability of methods used to assess connectivity, and on the true significance of the indices extrapolated from data (Reid et al., 2019).

Aim of this work is to assess the reliability of a non-linear measure (Transfer Entropy) used to investigate connectivity. Indeed, various recent papers underlined that TE represents an efficient method to estimate connectivity, which, compared with other methods, joins reliability and smaller computational time (Ito et al., 2011; Wang et al., 2014). Nevertheless, the relationship between TE, as a measure of information transfer, and the true anatomical connectivity between regions is still debated. In order to clarify this problem, we evaluated the significance of the connectivity values derived from bivariate TE by using the data generated through realistic models of neural populations coupled with assigned connectivity parameters. In particular, we investigated whether: (i) the method is able to discriminate between the presence or absence of connectivity between populations; (ii) the method is sensitive to a progressive change in the strength of the connection; (iii) the effects of non-linarites on TE estimation, in particular the effect of a change in populations working point; (iv) the effect of signal length and time delay. Moreover, all results have been compared with those obtained with the linear delayed correlation coefficient (see Supplementary Materials Part 2).

The importance of performing accurate validation studies for FC methods, based on simulation data, has been strongly emphasized in a recent perspective study by Reid et al. (2019). Both detailed neuron-level simulation models (Izhikevich, 2006; Goodman and Brette, 2008) or more abstract models, such as neural masses (Wendling et al., 2002; Sotero et al., 2007; Moran et al., 2008; Ursino et al., 2010; Bhattacharya et al., 2011; Cona et al., 2011, 2014; Cona and Ursino, 2015) can be of value to reach this objective, with alternative advantages and limitations. Our choice was to use NMMs which, as pointed out by Reid et al. (2019), see p. 1758 exhibit multiple advantages: among the others, computational efficiency, the possibility to generalize over multiple conditions, and the ease in the interpretation of results.

An important aspect to be recognized, however, is that NMMs are adequate to simulate (although with several approximations) the neuroelectrical activity in cortical columns, which may be significantly different from that measured on the scalp, due to propagation phenomena from the cortex to the skull through the interposed soft tissues. Hence, the present analysis images that the signals, obtained from scalp EEG/MEEG measurements, are first recreated on the cortex, via classic methods for source localization and reconstruction, before TE is calculated on them.

It is worth noting that the present study is focused on the bivariate algorithm for TE estimation [in particular, we used the open source toolbox Trentoool (Lindner et al., 2011), which is largely used in Neuroscience problems today]. The use of bivariate instead of multivariate TE surely represents the main limitation of the present study, and some of the errors encountered when simulating three or four populations (such as the presence of spurious connections) can be reduced using multivariate algorithms (such as those proposed in Montalto et al., 2014). This can be attempted in future works. For instance (Harmah et al., 2019) in a recent paper, evaluated multivariate TE in people with schizophrenia, and found that multivariate TE outperformed bivariate TE and Granger causality analysis under various signal-to-noise conditions. However, it is to be stressed that this difference between our results and those obtained with multivariate TE are probably not so strong as in other works, since Trentool implements some tools for *post-hoc* corrections of multivariate effects, i.e., a partial correction of spurious information flow.

Another limitation of the present approach is that we did not use other indices (like the “Coincidence Index,” see Shimono and Beggs, 2015) to improve the performance of our estimator. Indeed, the only additional measure we used is the DCC (see Supplementary Materials Part 2 and the last paragraph in the Discussion). Recently, Reid et al. (2019) suggested the simultaneous use of alternative measures, and their integration into a comprehensive framework, to improve connectivity estimate. This may be the subject of future work.

In order to realize physiologically reliable neural signals, with a frequency content analogous to that measured in cortical regions, we used the model proposed by the authors in recent years (Ursino et al., 2010). This allows multiple rhythms (for instance in the beta and gamma range) to be simultaneously produced and transmitted between regions, as a consequence of the non-linear feedback between excitatory and inhibitory populations (with glutamatergic, slow-GABAergic and fast-GABAergic synaptic dynamics). In particular, in this study we chose synaptic connections within the ROI [i.e., parameters C_ij_ in Equations (1)–(18)] to have power spectral densities quite similar to those occurring in pre-motor and supplementary cortical areas during motor tasks (Zavaglia et al., 2008, 2010).

It is worth noting that, in order to eliminate a possible bias in the estimation of TE, we always compared the TE value estimated from the model with that obtained on surrogate data (i.e., data with the same statistical properties of our signals but lacking of any connectivity). Hence, TE was set at zero whenever no statistical difference was observed between model signals and surrogate data; in all other cases, the (positive) difference between the model TE and that of surrogate data was assumed as an index of the synaptic strength.

(i) *Detection of spurious connections*—A first important result of our study is that the TE algorithm is able to discriminate between the presence of a significant connectivity, and the absence of connectivity rather well, in conditions when two ROIs are interconnected. In particular, in all cases when connectivity in the model was set at zero or at an extremely low value (W_P_ ≤ 10 or W_F_ ≤ 5), the algorithm provided no significant difference between model signals and surrogate data (Figures 3, 4, 10, 11). A similar result also holds when two populations receive a common signal from a third population, i.e., they have a common external source (Figure 5). Only in one case (Figure 5B left bottom panel) a very mild value of TE is obtained when the synapse W_P_ is zero. Conversely, in the more complex situations of Figure 6 (three interconnected ROIs), some artifacts can be seen in the computation of TE: we can observe a significant value of TE even when the corresponding synapse is zero. This is generally quite small, with the exception of the last snapshot in Figure 6C, when two synapses are at zero. It is worth noting that these “spurious” connections are always the consequence of a bi-synaptic link from the source region to the target one. Some spurious connections can be found, of course, also when simulating four interconnected ROIs (Figure 7), but their number remains quite limited. It may be interesting in future studies to test whether these “spurious” connectivity values can be eliminated or reduced by using multivariate methods for TE estimation. In particular, Olejarczyk et al. (2017) performed a comparison between the multivariate approach and the bivariate one for the analysis of effective connectivity in high density resting state EEG, and found that the multivariate approach is less sensitive to false indirect connections.

The previous results substantially agree with those by Wang et al. (2014) who, using signals obtained from NMMs with different connection strengths, observed that the bivariate TE provides high values of the Area under the ROC curves (i.e., a high measure of separability), hence the method performs very well in detecting the underlying connectivity structure. By the way, no evident difference was reported by Wang et al. (2014) when comparing the performance of the bivariate TE with that of the partial TE (see Figure 9 in their work).

(ii) *Dependence of TE estimation on synaptic strength*—An important result of our study is that quite a linear relationship can be observed between the estimated value of TE and the strength of the connection (either mono-synaptic excitatory or bi-synaptic inhibitory) in the same direction, provided the model is working in the linearity region. We are not aware of a similar analysis in the literature: indeed, most previous studies limit the investigation to the presence or absence of a connection (i.e., on its statistical significance, see also Vicente et al., 2011), or are based on ROC curves (see Wang et al., 2014). The linear relationship is quite straightforward in the case of excitatory synapses (Figure 3), and less precise in case of inhibitory synapses (Figure 4), but is still well evident when two populations are used. Furthermore, we also simulated conditions characterized by an excitation from ROI2 to ROI1 with a simultaneous inhibition from ROI1 to ROI2, and conditions in which ROI2 sends both an excitatory monosynaptic and a bi-synaptic inhibitory connection to ROI1. Sensitivity analysis on these cases, not reported from briefness, confirms what we observed in the other simulations: TE quite linearly depends on synapse strength, and inhibition in our model has a stronger effect than excitation.

In the more complex three-populations multivariate model (Figure 6) a clear positive correlation between TE and synapse strength is still evident, although influenced by the other synapse values. A very good correlation is still evident when using four interconnected ROIs (Figure 7) and, in this case, TE significantly outperforms the delayed correlation coefficient (see below). This result suggest that TE can be used (although with caution) not only to detect the presence or absence of a causal connection, but also to investigate whether this connection is stronger or weaker than another, or it is changing (reinforcing or weakening) with time. As commented below, however, a particular attention must be posed to any change in working conditions of a ROI (i.e., non linearity in the model), since it may affect TE dramatically.

Some authors recently emphasized that synapses among neurons exhibit a log-normal distribution (Song et al., 2005) and so that a few strong synapses dominate network dynamics over a large amount of weaker synapses. Although this result has been obtained in networks of hundreds of neurons (whereas our study is concerned with connections among a few ROIs) it may still be of interest in the problem of connectivity estimate, revealing that the main point is the capacity to estimate large synapses correctly (with minor emphasis on the smaller ones). Results in Figure 7 show that this aspect is well-managed by TE.

A further important result (although well-expected) is that TE cannot discriminate between an excitatory or a bi-synaptic inhibitory connection among two populations (we remind here that a inhibitory connection denotes a by-synaptic connection, from pre-synaptic pyramidal neurons to post-synaptic fast-GABAergic interneurons, and then to pyramidal neurons in the target population). This is quite obvious, since TE is always positive, and so it detects a sort of “absolute value” for the synaptic strength. In our model, inhibitory synapses are 2-fold more powerful in affecting signal transmission (hence TE) than excitatory connections. This result, however, depends on the parameters we used to simulate the internal number of synapses within populations. A different choice of internal parameters may modify this result. A similar conclusion (i.e., the incapacity to discriminate between excitatory and inhibitory connections via TE) has been reported in previous studies by considering synapses linking spiking neurons (Garofalo et al., 2009; Orlandi et al., 2014); we are not aware of a similar generalization considering the interactions among ROIs via NMMs simulations. However, as shown in Supplementary Materials Part 2, the delayed correlation coefficient is able to discriminate between excitatory and inhibitory connections very well. Hence, the simultaneous use of both metrics may allow this limitation to be easily overcome.

Finally, we wish to stress that the present study is devoted to the analysis of interactions among brain regions, and the mathematical model used to generate data simulates neural population dynamics; hence the results are not immediately applicable to the interactions among individual neurons.

(iii) *Effect of non-linearities*—A very important result of the present study is that TE strongly depends on the working point of the populations, and on the SD of the input noise. The first aspect is a consequence of the sigmoidal characteristics in the model, which describe the non-linear relationship linking post-synaptic membrane potential to spike density. In particular, whenever a population of pyramidal neurons enters into a saturation region, its capacity to transmit information toward other ROIs drastically decreases, despite the presence of a strong synapse. This is basically a consequence of a reduction in the entropy of the source signal (see Figures 8, 9, but see also Wollstadt et al. (2017) as an example of a reduction in source entropy induced by isofluorane anesthesia) although a significant exception can be found in one case in Figure 8. This aspect is crucial in the interpretation of TE, and has been clearly recognized by Wibral et al. (2014). These authors, when commenting on the relationship between TE and causality, underline the distinction between information transfer and causal interactions. In particular, they suggest that TE is not a measure of causal strength and that not all causal interactions serve the purpose to transmit information (Wibral et al., 2014, pp. 8–11). Hence, when using TE in the field of neuroscience, one must always take in mind that TE measures the amount of information that is transmitted from one region to another (or, in case of multivariate models, a certain amount of information transmitted through a bi-synaptic link). A high value of TE likely denotes the presence of a causal relationship, since information cannot be transmitted without coupling [care, however, should be taken in multivariate models to a shared information which may provide a spurious TE Timme and Lapish, 2018]. Conversely, a small value of TE does not necessarily indicates the absence of a causal link. Let us consider, for instance, the case in which activity in a pre-synaptic region shifts its working point reaching its upper saturation level (i.e., maximal activity of pyramidal neurons) and sends a strong synapse to a target population. Thanks to this coupling term, the second population can also enter into saturation (see for instance, Figure 8B leftmost panel and Figure 9B). At this point, TE is drastically reduced, and the appearance is that of poor information transmission. However, the first ROI may play an extremely important causal role on the second population even in this condition of poor information exchange. Let us consider, for instance, the case when a target region participates to a winner takes all dynamics against other regions: the causal link may be fundamental for it to win the competition, but is not detected (or just poorly detected) with the use of TE.

Moreover, TE is dramatically affected by the power level of input noise. In particular, it is not the level of noise *per se* which affects TE, but rather the relative contributions among the two populations. If noise to both populations rises together, TE does not increase, but rather exhibits a moderate reduction (Figure 8B rightmost panel). We attribute this decrease, evident only at very elevated power, to the presence of saturation in the sigmoid, which cut-offs the entropy of the source signals. Conversely, if one population receives much stronger noise than the other (Figure 8B middle panel), the amount of Entropy that it can transfer dramatically rises, while TE of the other is reduced, despite the presence of a similar reciprocal connectivity strength. Indeed, the first population can transmit much new information to the second, in the form of random fluctuations, while the information transmitted from the second to the first becomes quite negligible. In this particular case, we observed a surprising negative correlation between entropy in the first population (which decreases due to saturation) and TE it transfers to the second, that rather increases (see the bottom central panel in Figure 8B). This is an important point to be recognized in the interpretation of physiological results.

In conclusion, we can say that TE is quite linearly related with coupling strength as long as the populations work in the linear region and input noise is stable; this is no longer true if saturation is reached, or if other strong non-linear effects become influential (let us think, for instance, to synchronization in non-linear oscillators). Moreover, the estimated connectivity is strongly affected by the amount of activation that a population receives from randomized external sources. We think that this aspect has not been sufficiently investigated in previous studies using NMMs, where populations are used in linear working conditions and with stationary noise levels, and the non-linear effects on connectivity estimates are negligible. To confirm the possible disruptive effect of non-linearities on connectivity estimates, we remind the result by Wang et al. (2014): these authors observed that Granger causality and TE fail to discover the correct network topology when data are produced with strongly non-linear equations.

However, we wish to stress that in many neurocognitive problems estimation of TE may be of the greatest value even when its value is uncorrelated with the true causal connectivity: in fact, transfer of information may be more useful than measures of synaptic strength if the goal is to understand how the brain performs its computation and how one region transmit data to the other (see also Lizier and Prokopenko, 2010, as a nice illustration why transfer entropy may be more interesting when trying to understand a computation, compared to measures of physical causality). Indeed, the two measures are complementary, and knowledge of both may provide the best approach to the problem.

(iv) *The temporal duration and time delay*—Another important indication of the present study concerns the duration of the signals necessary to achieve quite a robust estimation of TE. This is an important point, since inconsistency in the length of the selected epochs can be found in the literature, which endangers a meaningful comparison between results. In particular, previous studies have shown that connectivity estimates are affected by the epoch length and that the severity of this bias varies for different connectivity metrics (David et al., 2004; Honey et al., 2007; Vinck et al., 2010; Chu et al., 2012; Bonita et al., 2014; Fraschini et al., 2016; Olejarczyk et al., 2017). Our results suggest that TE increases with signal duration, especially above 10 s (hence, caution should be taken when comparing experiments with different length). Conversely, the estimations with DCC are just scarcely affected by the signal length (Supplementary Materials Part 2). However, for signal lengths ≥10 s a clear linear relationship between TE and synaptic strength is evident, and an approximately linear relationship can still be detected for signal lengths of 3–4 s (Figure 11B). If lower durations are used (in particular, we used 2 s in Figure 11) TE fails to detect a clear relationship between TE and synapse strength (while DCC still offers good results): TE appears pretty high even at very low values of synaptic strength, and does not increase if the coupling term is increased. This effect of signal duration on connectivity estimation agrees with a few other results in the literature. Olejarczyk et al. (2017), who used multivariate TE for the analysis of connectivity in high density resting state EEG, used temporal windows as long as 20 s. Moreover, they observed that the smaller window which still ensures the quality of results is 10 s, and that the results do not change substantially if the epoch length is increased between 10 and 40 s; these results are quite comparable to our observations in Figure 11B. Fraschini et al. (2016) used two different measures of connectivity, i.e., the phase lag index (PLI) and the amplitude envelope correlation (AEC). Their results show that epoch length has an important impact on connectivity estimates: both mean PLI and AEC decrease with an increase in epoch length, with a tendency to stabilize at a length of 12 s for PLI and 6 s for AEC.

A final aspect concerns the time delay between signals. This is important in neuroscience, since spike transmission along axons in long-range connections can take several milliseconds to move from pre-synaptic to post-synaptic regions. Results in Figure 10 are quite unexpected, pointing out that TE estimation increases (and becomes more linear) with the time delay. This increase is evident also when using DCC. This result seems at odd with the results by Wang et al. (2014) who refer that TE was quite robust against variations of signal delay. However, the results of Wang et al. consider only the capacity to detect a given network topology, without investigating the relationship between TE and connectivity strength.

It is worth noting that, in our work, time delay is unknown to the algorithm, and is estimated within a given range assigned “a priori.” In particular, differences in Figure 10 cannot be significantly ascribed to an error in the evaluation of the time delay, since the values obtained by the algorithm (8, 15, and 25 ms) are not too distant from the real ones (10, 16.5, and 23 ms, respectively). If confirmed by other studies, this result may indicate that connectivity between proximal regions (assuming a smaller connection delay between them) can be somewhat underestimated compared with connectivity among more distal regions.

However, it is important to stress that, in many cases, an approximate value of the delay can be inferred from neurophysiologica/anatomical considerations, and this value should be used directly in the procedure. Indeed, we used a small range of values to drive Trentool algorithm. In the DCC estimates presented in Supplementary Materials Part 2, we always delayed the target signal by the number of samples closer to the true delay.

(v) *Comparison between TE and DCC*—All the estimates of functional connectivity obtained with TE have been replicated using the linear Delayed Correlation Coefficient, as shown in Supplementary Materials Part 2. A few conclusions can be drawn from this comparison. (a) TE provides a more reliable estimation of the connection strength. This is already evident when considering two ROIs connected in feedback, as in Figures 3, 4, 10, 11. In these cases, TE can recognize that one synapse remains constant while the other is varying, whereas the synapse strength estimated with DCC is significantly affected by a change in the other synapse. (b) TE works better than DCC in a multivariate network. This is especially evident comparing the values estimated using 4 interconnected ROIs (Figure 7), both for what concerns the correlation between the values estimated by the metrics and the model synapses strengths, and the number of spurious connections estimated by the metrics. (c) DCC is able to discriminate between excitatory and inhibitory connections, whereas TE provides only the absolute value of the connection. (d) DCC is less affected by noise, i.e., it exhibits a smaller standard deviation on repeated trials and less evident fluctuations. However, these differences are not so strong as to overcome differences noticed at point a. (e) The computational time is smaller for DCC than TE. (f) Both TE and DCC exhibit a similar behavior in response to non-linear changes, as examined in Figures 8, 9. In other terms, both evaluate a computational property, rather than a true causal connection.

As suggested by Reid et al. (2019) each metrics exhibits alternative virtues and limitations. The use of TE, integrated with a preliminary analysis with DCC, may represent a good approach to the study of network functional connectivity. DCC may provide a first rapid screening, able to discriminate between excitatory and inhibitory links, subsequently reinforced by a more accurate and reliable analysis with TE.

## Conclusions

In conclusion, using the open source toolbox Trentool, and neural mass models to generate biologically realistic signals, the present study provides indications on whether brain connectivity can be assessed from bivariate TE. In particular, we not only investigated whether the presence of a statistically significant connection can be detected (as in binary 0/1 network) but also if connection strength can be quantified. Results suggest that TE can be a promising method to estimate the strength of connectivity if neural populations work in the linear regions, and if the epoch lengths are longer than 10 s. In case of multivariate networks, some spurious connections can emerge (i.e., a statistically significant TE can be detected even in the absence of a true direct connection): however, quite a good correlation between TE and synaptic strength is still preserved in these cases, even when using four interconnected ROIs. A puzzling unexpected problem is the role of time delay: estimated TE appears higher for distal regions compared with proximal regions.

Finally, as well-expected, non-linear phenomena may play a dramatic role in the assessment of connectivity, since they may significantly reduce the estimation of TE. In fact, TE is an index of information transfer and not directly an index of connectivity strength. In particular, due to non-linear relationships between the connected regions, a strong causal strength may be present between two nodes in a network, even if the detected TE is very small. We claim that similar problems can be found not only with TE but also if other metrics of connectivity (in particular those based on autoregressive models) are used, as shown when using the Delayed Correlation Coefficient. This is perhaps the most important aspect of the present work, which deserves accurate *ad hoc* investigation. We suggest that changes in connectivity, often reported in the literature during different tasks, or in different brain conditions, might not always reflect a true change in the connecting network, but rather a change in information transmission due to a different working region of the involved populations. However, in conditions when linearity is a good approximation of the system, changes in TE can actually reflect true changes in connectivity. Hence, researchers need to carefully consider non-linearity to apply bivariate TE. Moreover, they should check bivariate vs. multivariate TE to improve their estimation.

## Data Availability Statement

The neural mass model has been implemented in Matlab. Matlab codes and data are available in ModelDB (A neural mass model for critical assessment of brain connectivity; Ursino et al., 2020) at http://modeldb.yale.edu/263637. In these codes the author provide the program for generating the data, an example of the code for TE computation from data, and instructions on how to replicate each case. Hence, all datasets (simulating local field potentials and used for TE estimation) can be generated from the Matlab codes. The Trentool Matlab toolbox for Transfer Entropy estimation is open source and can be download at http://trentool.github.io/TRENTOOL3/.

## Author Contributions

MU conceived and designed the research, developed the neural mass model, analyzed and interpreted the results, drafted, and edited the manuscript. GR contributed to research conception performed simulations, analyzed, and interpreted the results. EM contributed to research conception analyzed and interpreted the results, revised, and edited the manuscript.

## Conflict of Interest

The authors declare that the research was conducted in the absence of any commercial or financial relationships that could be construed as a potential conflict of interest.

## References

[B1] Ansari-Asl K. Senhadji L. Bellanger J.-J. Wendling F. (2006). Quantitative evaluation of linear and nonlinear methods characterizing interdependencies between brain signals. Phys. Rev. E Stat. Nonlin. Soft Matter Phys. 74:031916. 10.1103/PhysRevE.74.031916 17025676 PMC2071949

[B2] Astolfi L. de Vico Fallani F. Cincotti F. Mattia D. Marciani M. G. Bufalari S.. (2007). Imaging functional brain connectivity patterns from high-resolution EEG and fMRI via graph theory. Psychophysiology 44, 880–893. 10.1111/j.1469-8986.2007.00556.x 17617172

[B3] Bajaj S. Adhikari B. M. Friston K. J. Dhamala M. (2016). Bridging the gap: dynamic causal modeling and granger causality analysis of resting state functional magnetic resonance imaging. Brain Connect 6, 652–661. 10.1089/brain.2016.0422 27506256

[B4] Bastos A. M. Schoffelen J.-M. (2015). A tutorial review of functional connectivity analysis methods and their interpretational pitfalls. Front. Syst. Neurosci. 9:175. 10.3389/fnsys.2015.00175 26778976 PMC4705224

[B5] Bhattacharya B. S. Coyle D. Maguire L. P. (2011). A thalamo-cortico-thalamic neural mass model to study alpha rhythms in Alzheimer's disease. Neural Netw. 24, 631–645. 10.1016/j.neunet.2011.02.009 21435838

[B6] Bonita J. D. Ambolode L. C. C. Rosenberg B. M. Cellucci C. J. Watanabe T. A. A. Rapp P. E.. (2014). Time domain measures of inter-channel EEG correlations: a comparison of linear, nonparametric and nonlinear measures. Cogn. Neurodyn. 8, 1–15. 10.1007/s11571-013-9267-8 24465281 PMC3890093

[B7] Chu C. J. Kramer M. A. Pathmanathan J. Bianchi M. T. Westover M. B. Wizon L.. (2012). Emergence of stable functional networks in long-term human electroencephalography. J. Neurosci. 32, 2703–2713. 10.1523/JNEUROSCI.5669-11.2012 22357854 PMC3361717

[B8] Cona F. Lacanna M. Ursino M. (2014). A thalamo-cortical neural mass model for the simulation of brain rhythms during sleep. J. Comput. Neurosci. 37, 125–148. 10.1007/s10827-013-0493-1 24402459

[B9] Cona F. Ursino M. (2015). A neural mass model of place cell activity: theta phase precession, replay and imagination of never experienced paths. J. Comput. Neurosci. 38, 105–127. 10.1007/s10827-014-0533-5 25284339

[B10] Cona F. Zavaglia M. Massimini M. Rosanova M. Ursino M. (2011). A neural mass model of interconnected regions simulates rhythm propagation observed via TMS-EEG. Neuroimage 57, 1045–1058. 10.1016/j.neuroimage.2011.05.007 21600291

[B11] David O. Cosmelli D. Friston K. J. (2004). Evaluation of different measures of functional connectivity using a neural mass model. Neuroimage 21, 659–673. 10.1016/j.neuroimage.2003.10.006 14980568

[B12] David O. Friston K. J. (2003). A neural mass model for MEG/EEG: coupling and neuronal dynamics. Neuroimage 20, 1743–1755. 10.1016/j.neuroimage.2003.07.015 14642484

[B13] David O. Harrison L. Friston K. J. (2005). Modelling event-related responses in the brain. Neuroimage 25, 756–770. 10.1016/j.neuroimage.2004.12.030 15808977

[B14] Felleman D. J. Van Essen D. C. (1991). Distributed hierarchical processing in the primate cerebral cortex. Cereb. Cortex 1, 1–47. 10.1093/cercor/1.1.1 1822724

[B15] Fraschini M. Demuru M. Crobe A. Marrosu F. Stam C. J. Hillebrand A. (2016). The effect of epoch length on estimated EEG functional connectivity and brain network organisation. J. Neural Eng. 13:036015. 10.1088/1741-2560/13/3/036015 27137952

[B16] Friston K. (2009). Causal modelling and brain connectivity in functional magnetic resonance imaging. PLoS Biol. 7:e33 10.1371/journal.pbio.1000033 19226186 PMC2642881

[B17] Friston K. Moran R. Seth A. K. (2013). Analysing connectivity with Granger causality and dynamic causal modelling. Curr. Opin. Neurobiol. 23, 172–178. 10.1016/j.conb.2012.11.010 23265964 PMC3925802

[B18] Garofalo M. Nieus T. Massobrio P. Martinoia S. (2009). Evaluation of the performance of information theory-based methods and cross-correlation to estimate the functional connectivity in cortical networks. PLoS ONE 4:e6482. 10.1371/journal.pone.0006482 19652720 PMC2715865

[B19] Goodman D. Brette R. (2008). Brian: a simulator for spiking neural networks in python. Front. Neuroinform. 2:5. 10.3389/neuro.11.005.2008 19115011 PMC2605403

[B20] Granger C. W. J. (1980). Long memory relationships and the aggregation of dynamic models. J. Econometr. 14, 227–238. 10.1016/0304-4076(80)90092-5

[B21] Grefkes C. Eickhoff S. B. Nowak D. A. Dafotakis M. Fink G. R. (2008). Dynamic intra- and interhemispheric interactions during unilateral and bilateral hand movements assessed with fMRI and DCM. Neuroimage 41, 1382–1394. 10.1016/j.neuroimage.2008.03.048 18486490

[B22] Harmah D. J. Li C. Li F. Liao Y. Wang J. Ayedh W. M. A.. (2019). Measuring the non-linear directed information flow in schizophrenia by multivariate transfer entropy. Front. Comput. Neurosci. 13:85. 10.3389/fncom.2019.00085 31998105 PMC6966771

[B23] Honey C. J. Kötter R. Breakspear M. Sporns O. (2007). Network structure of cerebral cortex shapes functional connectivity on multiple time scales. Proc. Natl. Acad. Sci. U.S.A. 104, 10240–10245. 10.1073/pnas.0701519104 17548818 PMC1891224

[B24] Horwitz B. (2003). The elusive concept of brain connectivity. Neuroimage 19, 466–470. 10.1016/S1053-8119(03)00112-5 12814595

[B25] Ito S. Hansen M. E. Heiland R. Lumsdaine A. Litke A. M. Beggs J. M. (2011). Extending transfer entropy improves identification of effective connectivity in a spiking cortical network model. PLoS ONE 6:e27431. 10.1371/journal.pone.0027431 22102894 PMC3216957

[B26] Izhikevich E. M. (2006). Polychronization: computation with spikes. Neural Comput. 18, 245–282. 10.1162/089976606775093882 16378515

[B27] Koenig T. Studer D. Hubl D. Melie L. Strik W. K. (2005). Brain connectivity at different time-scales measured with EEG. Philos. Trans. R. Soc. Lond. B Biol. Sci. 360, 1015–1023. 10.1098/rstb.2005.1649 16087445 PMC1854932

[B28] Lindner M. Vicente R. Priesemann V. Wibral M. (2011). TRENTOOL: a Matlab open source toolbox to analyse information flow in time series data with transfer entropy. BMC Neurosci. 12:119. 10.1186/1471-2202-12-119 22098775 PMC3287134

[B29] Lizier J. T. Prokopenko M. (2010). Differentiating information transfer and causal effect. Eur. Phys. J. B 73, 605–615. 10.1140/epjb/e2010-00034-5

[B30] Lobier M. Siebenhühner F. Palva S. Palva J. M. (2014). Phase transfer entropy: a novel phase-based measure for directed connectivity in networks coupled by oscillatory interactions. Neuroimage 85(Pt 2), 853–872. 10.1016/j.neuroimage.2013.08.056 24007803

[B31] Mangia A. L. Ursino M. Lannocca M. Cappello A. (2017). Transcallosal Inhibition during motor imagery: Analysis of a Neural Mass Model. Front. Comput. Neurosci. 11:57. 10.3389/fncom.2017.00057 28713259 PMC5491977

[B32] Montalto A. Faes L. Marinazzo D. (2014). MuTE: a MATLAB toolbox to compare established and novel estimators of the multivariate transfer entropy. PLoS ONE 9:e109462. 10.1371/journal.pone.0109462 25314003 PMC4196918

[B33] Moran R. J. Stephan K. E. Kiebel S. J. Rombach N. O'Connor W. T. Murphy K. J.. (2008). Bayesian estimation of synaptic physiology from the spectral responses of neural masses. Neuroimage 42, 272–284. 10.1016/j.neuroimage.2008.01.025 18515149 PMC2644419

[B34] Nichols J. M. Seaver M. Trickey S. T. Todd M. D. Olson C. Overbey L. (2005). Detecting nonlinearity in structural systems using the transfer entropy. Phys. Rev. E Stat. Nonlin. Soft Matter Phys. 72:046217. 10.1103/PhysRevE.72.046217 16383522

[B35] Olejarczyk E. Marzetti L. Pizzella V. Zappasodi F. (2017). Comparison of connectivity analyses for resting state EEG data. J. Neural Eng. 14:036017. 10.1088/1741-2552/aa6401 28378705

[B36] Orlandi J. G. Stetter O. Soriano J. Geisel T. Battaglia D. (2014). Transfer entropy reconstruction and labeling of neuronal connections from simulated calcium imaging. PLoS ONE 9:e98842. 10.1371/journal.pone.0098842 24905689 PMC4048312

[B37] Penny W. D. Stephan K. E. Mechelli A. Friston K. J. (2004). Comparing dynamic causal models. Neuroimage 22, 1157–1172. 10.1016/j.neuroimage.2004.03.026 15219588

[B38] Pool E.-M. Leimbach M. Binder E. Nettekoven C. Eickhoff S. B. Fink G. R.. (2018). Network dynamics engaged in the modulation of motor behavior in stroke patients. Hum. Brain Mapp. 39, 1078–1092. 10.1002/hbm.23872 29193484 PMC5807219

[B39] Reid A. T. Headley D. B. Mill R. D. Sanchez-Romero R. Uddin L. Q. Marinazzo D.. (2019). Advancing functional connectivity research from association to causation. Nat. Neurosci. 22, 1751–1760. 10.1038/s41593-019-0510-4 31611705 PMC7289187

[B40] Rossini P. M. Di Iorio R. Bentivoglio M. Bertini G. Ferreri F. Gerloff C.. (2019). Methods for analysis of brain connectivity: An IFCN-sponsored review. Clin. Neurophysiol. 130, 1833–1858. 10.1016/j.clinph.2019.06.006 31401492

[B41] Sakkalis V. (2011). Review of advanced techniques for the estimation of brain connectivity measured with EEG/MEG. Comput. Biol. Med. 41, 1110–1117. 10.1016/j.compbiomed.2011.06.020 21794851

[B42] Schindler K. Palus M. Vejmelka M. Bhattacharya J. (2007). Causality detection based on information-theoretic approaches in time series analysis. Phys. Rep. 441, 1–46. 10.1016/j.physrep.2006.12.004

[B43] Schreiber T. (2000). Measuring information transfer. Phys. Rev. Lett. 85, 461–464. 10.1103/PhysRevLett.85.461 10991308

[B44] Shimono M. Beggs J. M. (2015). Functional clusters, hubs, and communities in the cortical microconnectome. Cereb. Cortex 25, 3743–3757. 10.1093/cercor/bhu252 25336598 PMC4585513

[B45] Song S. Sjöström P. J. Reigl M. Nelson S. Chklovskii D. B. (2005). Highly nonrandom features of synaptic connectivity in local cortical circuits. PLoS Biol. 3:e68. 10.1371/journal.pbio.0030068 15737062 PMC1054880

[B46] Sotero R. C. Trujillo-Barreto N. J. Iturria-Medina Y. Carbonell F. Jimenez J. C. (2007). Realistically coupled neural mass models can generate EEG rhythms. Neural Comput. 19, 478–512. 10.1162/neco.2007.19.2.478 17206872

[B47] Sporns O. (2011). The human connectome: a complex network. Ann. N. Y. Acad. Sci. 1224, 109–125. 10.1111/j.1749-6632.2010.05888.x 21251014

[B48] Sporns O. (2013). Network attributes for segregation and integration in the human brain. Curr. Opin. Neurobiol. 23, 162–171. 10.1016/j.conb.2012.11.015 23294553

[B49] Takens F. (1980). “Dynamical systems and turbulence,” in Lecture Notes in Mathematics. Detecting Strange Attractors in Turbulence, eds D. Rand and L. S. Young (New York, NY Springer-Verlag), Vol. 898, 366–381.

[B50] Timme N. M. Lapish C. (2018). A tutorial for information theory in neuroscience. eNeuro 5. 10.1523/ENEURO.0052-18.2018 30211307 PMC6131830

[B51] Ursino M. Cona F. Zavaglia M. (2010). The generation of rhythms within a cortical region: analysis of a neural mass model. Neuroimage 52, 1080–1094. 10.1016/j.neuroimage.2009.12.084 20045071

[B52] Valdes-Sosa P. A. Roebroeck A. Daunizeau J. Friston K. (2011). Effective connectivity: influence, causality and biophysical modeling. Neuroimage 58, 339–361. 10.1016/j.neuroimage.2011.03.058 21477655 PMC3167373

[B53] van den Heuvel M. P. Hulshoff Pol H. E. (2010). Exploring the brain network: a review on resting-state fMRI functional connectivity. Eur. Neuropsychopharmacol. 20, 519–534. 10.1016/j.euroneuro.2010.03.008 20471808

[B54] Vicente R. Wibral M. Lindner M. Pipa G. (2011). Transfer entropy–a model-free measure of effective connectivity for the neurosciences. J. Comput. Neurosci. 30, 45–67. 10.1007/s10827-010-0262-3 20706781 PMC3040354

[B55] Vinck M. van Wingerden M. Womelsdorf T. Fries P. Pennartz C. M. A. (2010). The pairwise phase consistency: a bias-free measure of rhythmic neuronal synchronization. Neuroimage 51, 112–122. 10.1016/j.neuroimage.2010.01.073 20114076

[B56] Wang H. E. Bénar C. G. Quilichini P. P. Friston K. J. Jirsa V. K. Bernard C. (2014). A systematic framework for functional connectivity measures. Front. Neurosci. 8:405. 10.3389/fnins.2014.00405 25538556 PMC4260483

[B57] Wendling F. Ansari-Asl K. Bartolomei F. Senhadji L. (2009). From EEG signals to brain connectivity: a model-based evaluation of interdependence measures. J. Neurosci. Methods 183, 9–18. 10.1016/j.jneumeth.2009.04.021 19422854

[B58] Wendling F. Bartolomei F. Bellanger J. J. Chauvel P. (2002). Epileptic fast activity can be explained by a model of impaired GABAergic dendritic inhibition. Eur. J. Neurosci. 15, 1499–1508. 10.1046/j.1460-9568.2002.01985.x 12028360

[B59] Wibral M. Pampu N. Priesemann V. Siebenhühner F. Seiwert H. Lindner M.. (2013). Measuring information-transfer delays. PLoS ONE 8:e55809. 10.1371/journal.pone.0055809 23468850 PMC3585400

[B60] Wibral M. Vicente R. Lindner M. (2014). “Transfer entropy in neuroscience,” in Directed Information Measures in Neuroscience Understanding Complex Systems, eds M. Wibral, R. Vicente, and J. T. Lizier (Berlin: Springer Berlin Heidelberg), 3–36.

[B61] Wiener N. (1956). “The theory of prediction,” in Modern Mathematics for the Engineer, eds E. F. Beckenbach and R. Weller (New York, NY: McGraw-Hill), 165–190.

[B62] Wollstadt P. Martínez-Zarzuela M. Vicente R. Díaz-Pernas F. J. Wibral M. (2014). Efficient transfer entropy analysis of non-stationary neural time series. PLoS ONE 9:e102833. 10.1371/journal.pone.0102833 25068489 PMC4113280

[B63] Wollstadt P. Sellers K. K. Rudelt L. Priesemann V. Hutt A. Fröhlich F.. (2017). Breakdown of local information processing may underlie isoflurane anesthesia effects. PLoS Comput. Biol. 13:e1005511. 10.1371/journal.pcbi.1005511 28570661 PMC5453425

[B64] Zavaglia M. Astolfi L. Babiloni F. Ursino M. (2008). The effect of connectivity on EEG rhythms, power spectral density and coherence among coupled neural populations: analysis with a neural mass model. IEEE Trans. Biomed. Eng. 55, 69–77. 10.1109/TBME.2007.897814 18232348

[B65] Zavaglia M. Cona F. Ursino M. (2010). A neural mass model to simulate different rhythms in a cortical region. Comput. Intell. Neurosci. 2010:456140. 10.1155/2010/456140 20037742 PMC2796462

