# Supplementary material for: Transfer Entropy as a Measure of Brain Connectivity: A Critical Analysis With the Help of Neural Mass Models
Source: Front Comput Neurosci. 2020 Jun 5;14:45. doi: 10.3389/fncom.2020.00045 (PMC7292208; doi:10.3389/fncom.2020.00045)
Supplement: Supplementary file 1 [file Data_Sheet_1.PDF]

Supplementary Materials part 1:

Parameters of Trentool adopted

in the present analysis

TRANSFER ENTROPY AS A MEASURE OF BRAIN CONNECTIVITY: A CRITICAL ANALYSIS WITH THE HELP OF NEURAL MASS MODELS

Mauro Ursino*, Giulia Ricci, Elisa Magosso

Department of Electrical, Electronic and Information Engineering, University of Bologna, Campus of Cesena, Cesena, Italy.

In the following, the version of Trentool used and a list of all parameters adopted is described. The readers can refer to the manual documentation: TRENTOOL 3.4.0 beta – User Documentation by Patricia Wollstadt, Michael Lindner, Raul Vicente Michael Wibral, Nicu Pampu and Mario Martinez-Zarzuela, Version 0.93, http://www.trentool.de/ for more details.

TRENTOOL 3.4.0 beta

Version 0.93

Table 1

Parameters for the configuration structure cfgTEP. of the functions TEprepare and InteractionDelayReconstruction_calculate (TRENTOOL Version 3.4)

Field Name	Data Type	Value	Description
TEcalctype	string	‘VM_ds’	Estimator guaranteeing optimal self-prediction
predictime_u	Integer (ms)	15	Assumed information transfer delay *u* between source and target time series
predicttimemax_u	Integer (ms)	18	Maximum u to be scanned
predicttimemin_u	Integer (ms)	12	Minimum u to be scanned
predicttimestepsize	integer	1	Time steps between u’s to be scanned
ensemblemethod	string	’no’	Use of the ensemble-method for (time-resolved) TE estimation
kth_neighbors	integer	4	Number of neighbours for fixed mass search (controls balance of bias/statistical errors)
TheilerT	string	’ACT’	Number of temporal neighbours excluded to avoid serial correlations (Theiler correction)
maxlag	Integer (samples)	1000	The range of lags for computing the ACT: from - MAXLAG to MAXLAG
trialselect	string	‘no’	Sets a minimum number of trials that have to survive trial selection
actthrvalue	integer	30	Max threshold for the ACT for trial selection
optimizemethod	string	‘ragwitz’	Define method for parameter optimization: ’ragwitz’
verbosity	string	’info_minor’	Defines the verbosity of console output of TRENTOOL
ragdim	integer	4:8	For Ragwitz: range of embedding dimensions to scan vector from 1 to n
ragtaurange	double	[0.8 1.8]	For Ragwitz: 1x2-vector of min and max embedding delays
ragtausteps	integer	10	For Ragwitz: number of equidistant steps in ragtaurange with a minimum of 5
flagNei	string	‘Mass’	For Ragwitz: ’Range’ or ’Mass’ type of neighbor search
sizeNei	integer	4	For Ragwitz: Radius or mass for the neighbor search according to flagNei
repPred	integer	100	For Ragwitz: repPred represents the number of sample points for which the prediction is performed

Table 2

Parameters for the configuration structure cfgTESS of the functions TEsurrogatestats and InteractionDelayReconstruction_calculate (TRENTOOL Version 3.4)

Field Name	Data Type	Value	Description
optdimusage	string	'indivdim'	’indivdim’ to use the individual optimal dimension for each channel
dim	integer	Output TEprepare	Value(s) for embedding dimension. This is automatically taken from the field TEprepare in the data
tau	integer	Output TEprepare	Embedding delay in units of act (x*act). This is automatically taken from the field TEprepare in the data
alpha	double	0.05	Significance level for statisatical permutation test
tail	integer	1	1 tail test of significance (for the permutation tests)
surrogatetype	string	'trialshuffling'	Strategy for surrogate data creation
extracond	string	'Faes_Method'	Perform conditioning in tansfer entropy formula on additional variables. Values: ’Faes_Method’
shifttest	string	'no'	’yes’ string Perform shift test to identify instantaneous mixing between the signal pairs.
MIcalc	integer	1	Determines whether mutual information is calculated additionally to TE (1) or not (0)
shifttesttype	string	’TE>TEshift’	The shift test can be calculated for the direction TE value of original data > TE values of shifted data (value = ’TE>TEshift’)
shifttype	string	’predicttime’	Shifting the length of the ’predicttime’
numpermutation	integer	190100/500a	Nr of permutations in permutation test
permstatstype	string	’indepsamplesT	Type of the test statistic used: ‘indepsamplesT’ for distribution of the t-values
correctm	string	’FDR’	Correction method used for correction of the multiple comparison problem over all analyzed channel combinations - False discovery rate ’FDR’
