# Supplementary material for: Transfer Entropy as a Measure of Brain Connectivity: A Critical Analysis With the Help of Neural Mass Models
Source: Front Comput Neurosci. 2020 Jun 5;14:45. doi: 10.3389/fncom.2020.00045 (PMC7292208; doi:10.3389/fncom.2020.00045)
Supplement: Supplementary file 2 [file Data_Sheet_2.PDF]

Supplementary Materials Part 2:

Analysis with the Delayed Correlation Coefficient

TRANSFER ENTROPY AS A MEASURE OF BRAIN CONNECTIVITY: A CRITICAL ANALYSIS WITH THE HELP OF NEURAL MASS MODELS

Mauro Ursino*, Giulia Ricci, Elisa Magosso

Department of Electrical, Electronic and Information Engineering, University of Bologna, Campus of Cesena, Cesena, Italy.

In this Supplementary material, we provide the results obtained with the same analysis presented in the Manuscript, but using the delayed correlation coefficient (DCC) instead of Transfer Entropy. In particular, we computed the pairwise linear correlation coefficient between the source signal and the delayed target signal. The delay was chosen equal to two sampling periods (20 ms) for all tests. Only in Fig. 10, we used a delay as low as 1 sampling period (10 ms) for the left panel, and 3 sampling periods (30 ms) for the right panel.

All figures have the same meaning as the figures shown in the text. The only difference is that, for clarity, we show the results ± SD (instead of ± SEM) since SEM is very small when computed on DCC estimates, and so it would be scarcely visible in the figures. We just remind that, in our tests, we have SEM = SD / $\sqrt{10}$.

Figure 3S

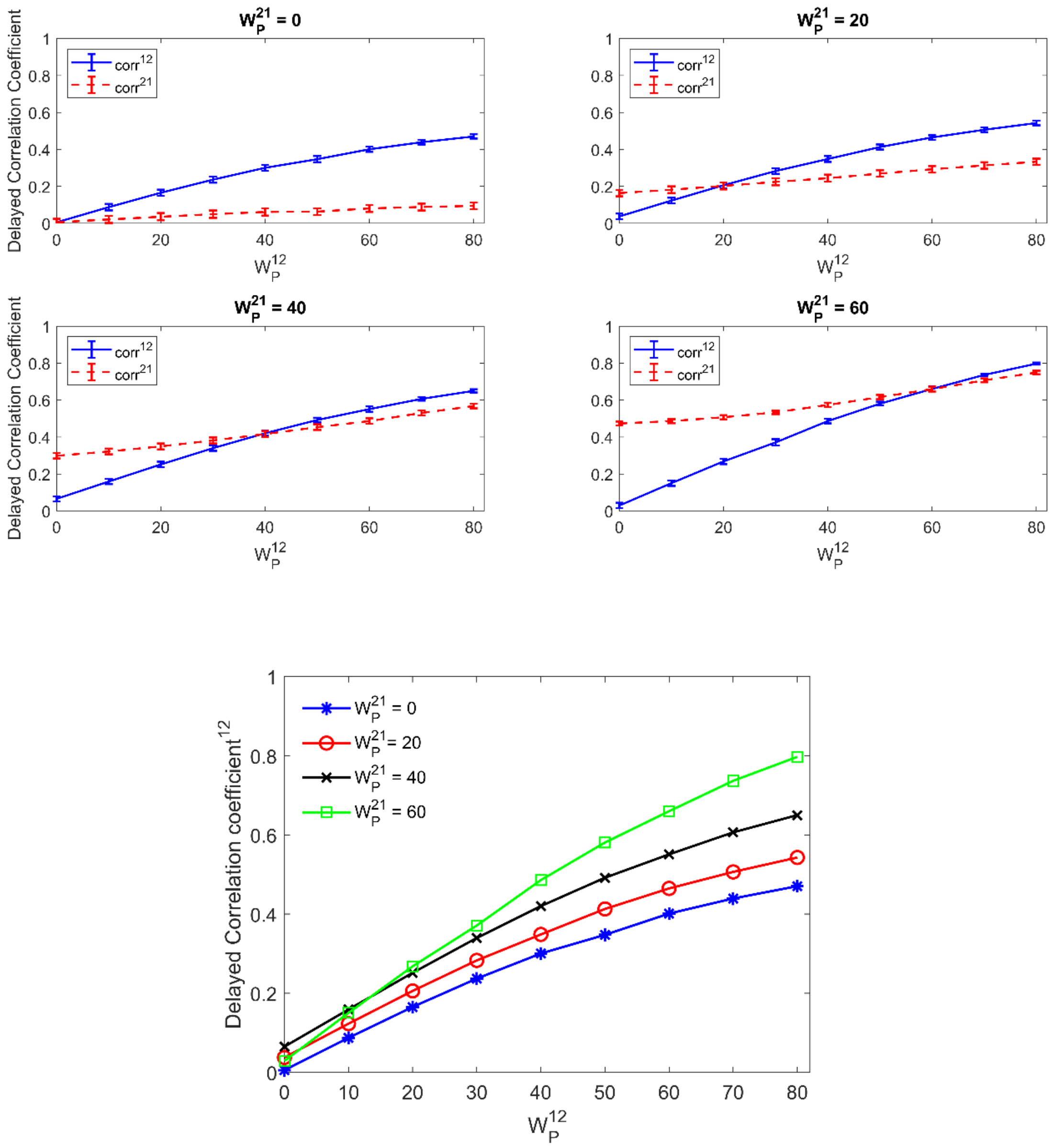

Figure 3S – Dependence of the delayed correlation coefficient (DCC) on a feedback, realized assuming two regions interconnected with reciprocal excitatory synapses (upper panels). See text for more details

Figure 4S

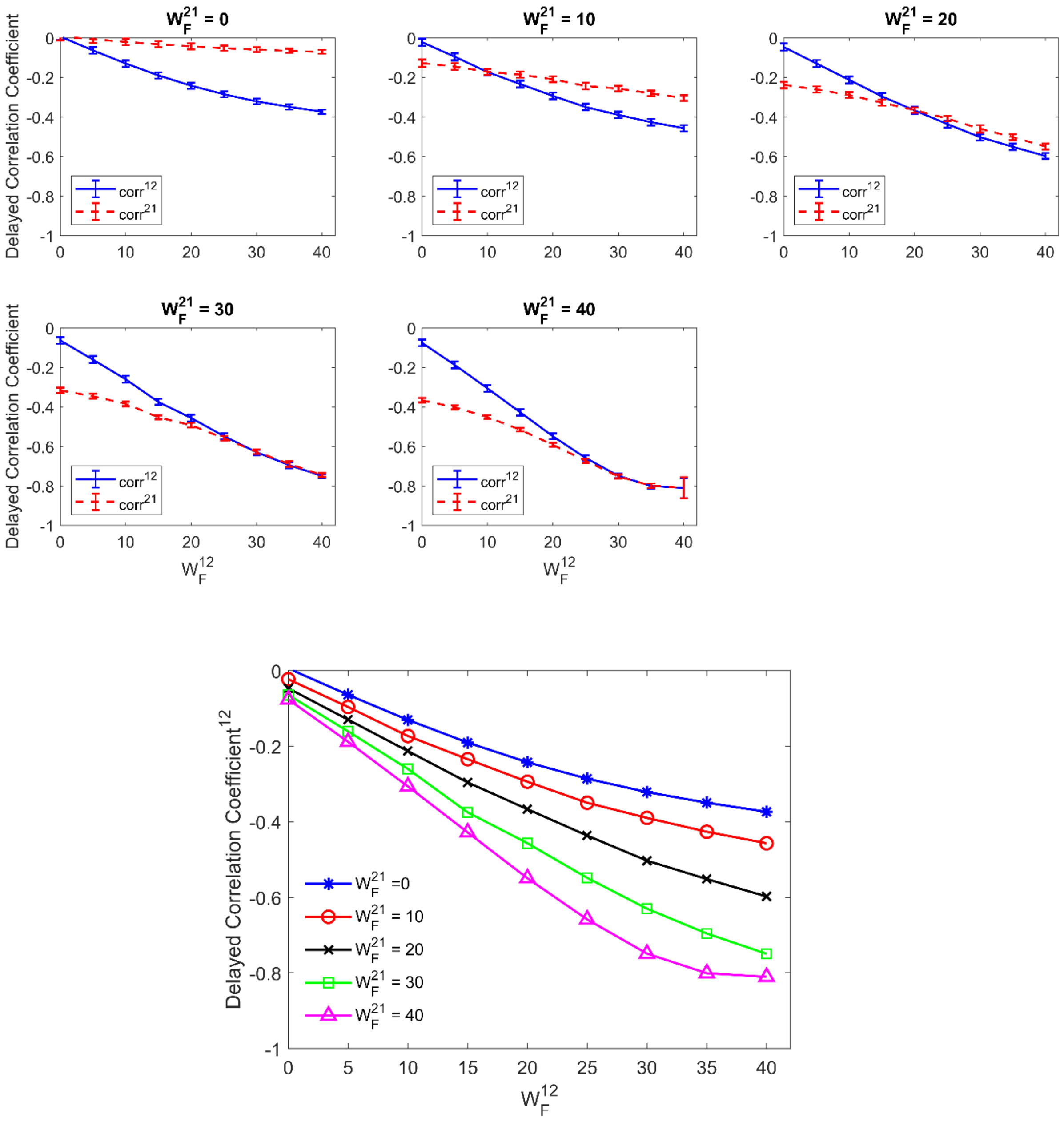

Figure 4S – Dependence of the delayed correlation coefficient (DCC) on a feedback realized assuming two regions interconnected with reciprocal inhibitory synapses. See text for more details.

Figure 5S

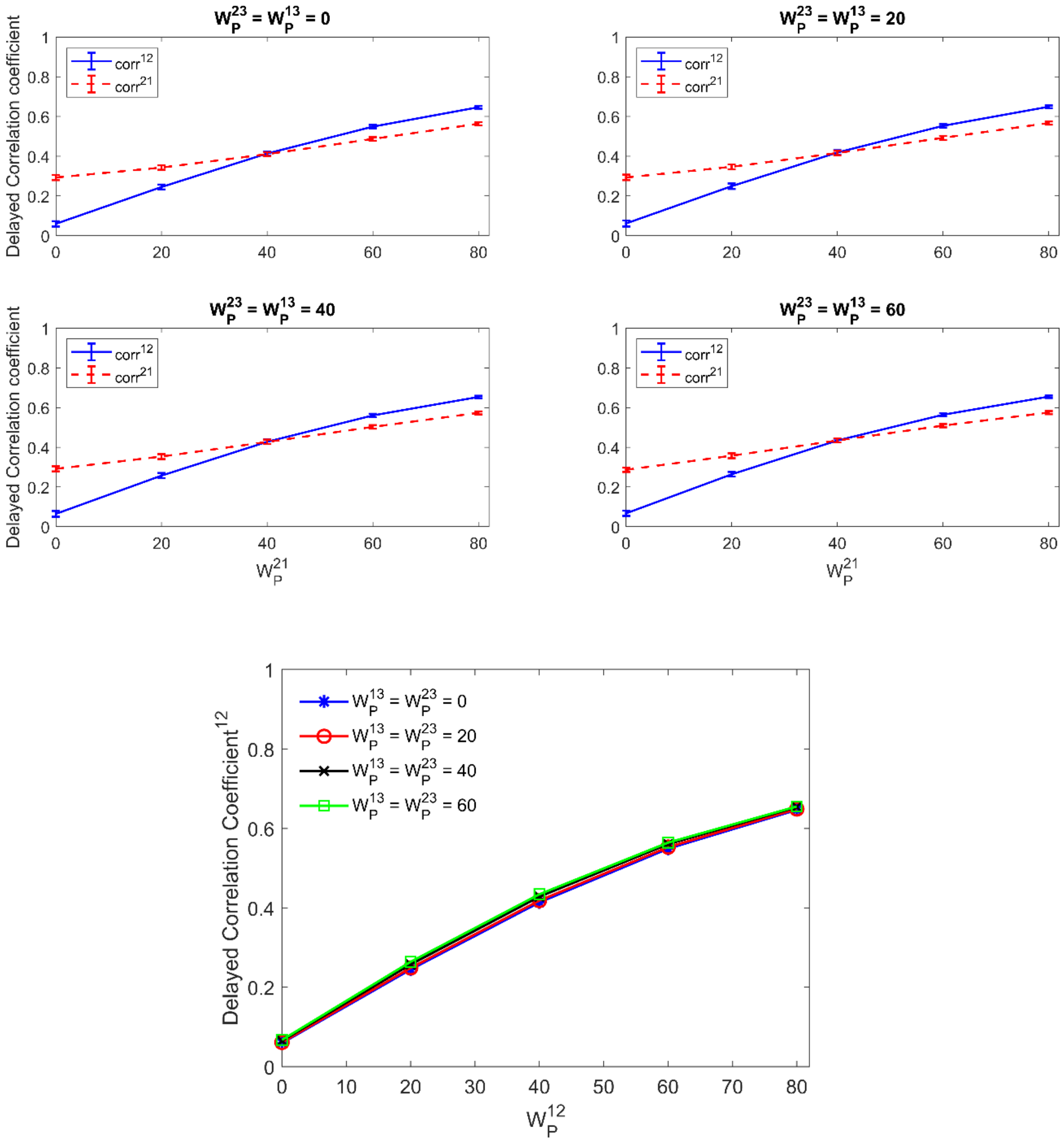

Figure 5S – Influence of a common external source on DCC estimation. Simulations were performed assuming three regions interconnected via an excitatory synapse from region 2 to region 1, which was progressively varied between 0 and 80, and a constant excitatory synapse in the other direction set at a constant value. See text for more details.

Figure 6S

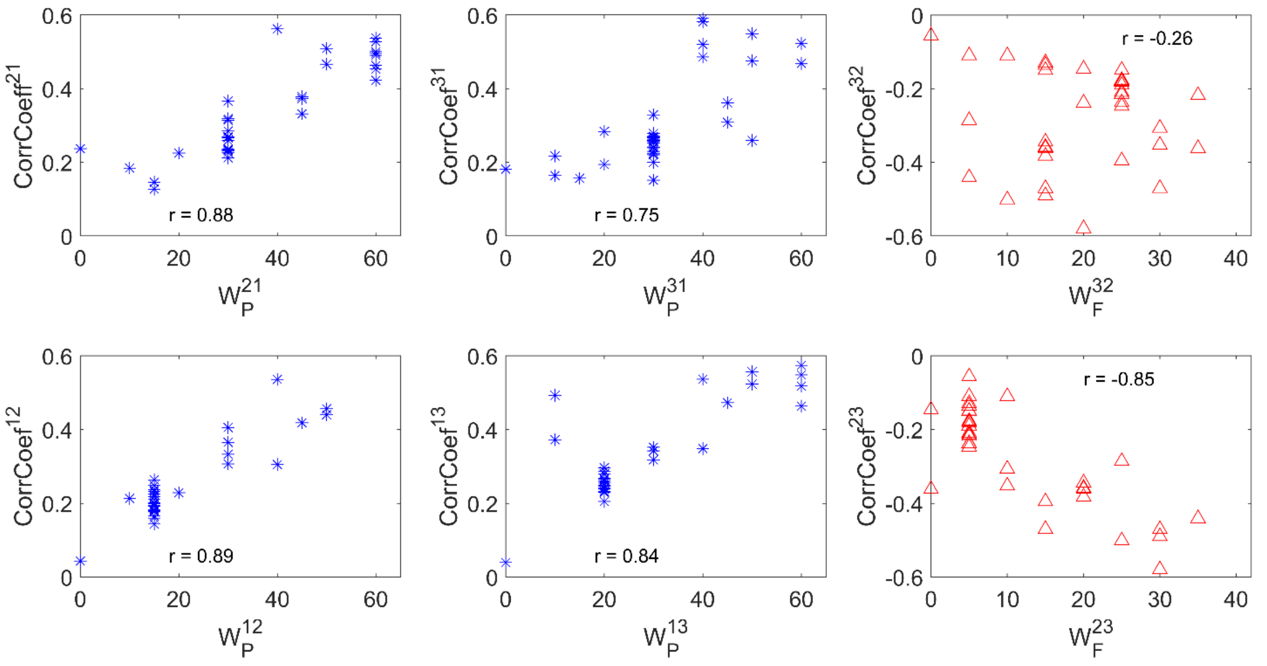

Figure 6S – Effect of different combinations of synapses on DCC in a model of three interconnected regions, where regions 2 and 3 are in competition via inhibitory synapses and are linked via excitatory synapses to region 1. All other synapses are set at zero. See text for more details.

Figure 7S

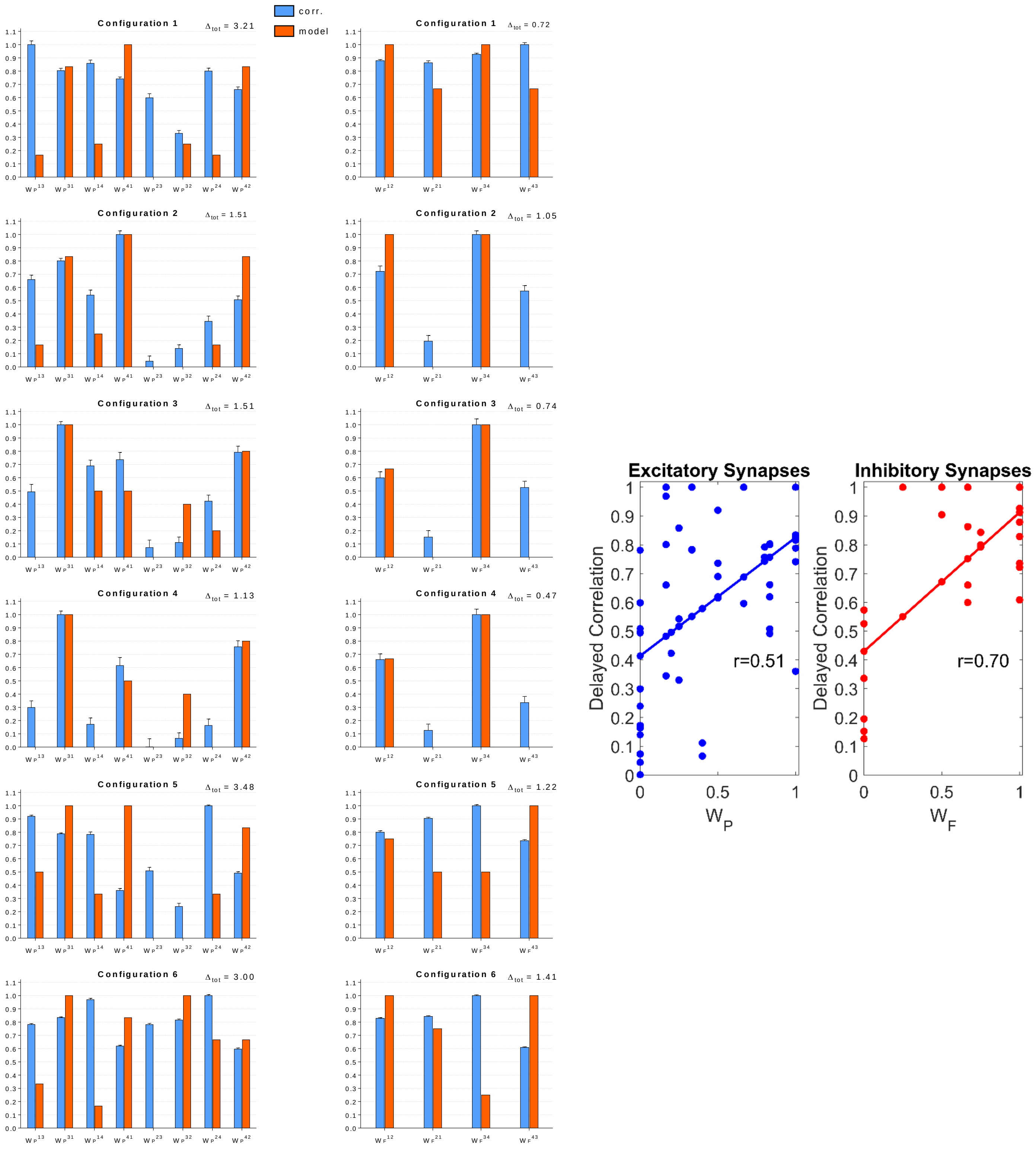

Figure 7S – Estimation of the connectivity strength with DCC obtained during six different simulations, each performed with four interconnected ROIs. Each row refers to a different network configuration. See text for details.

Figure 8S

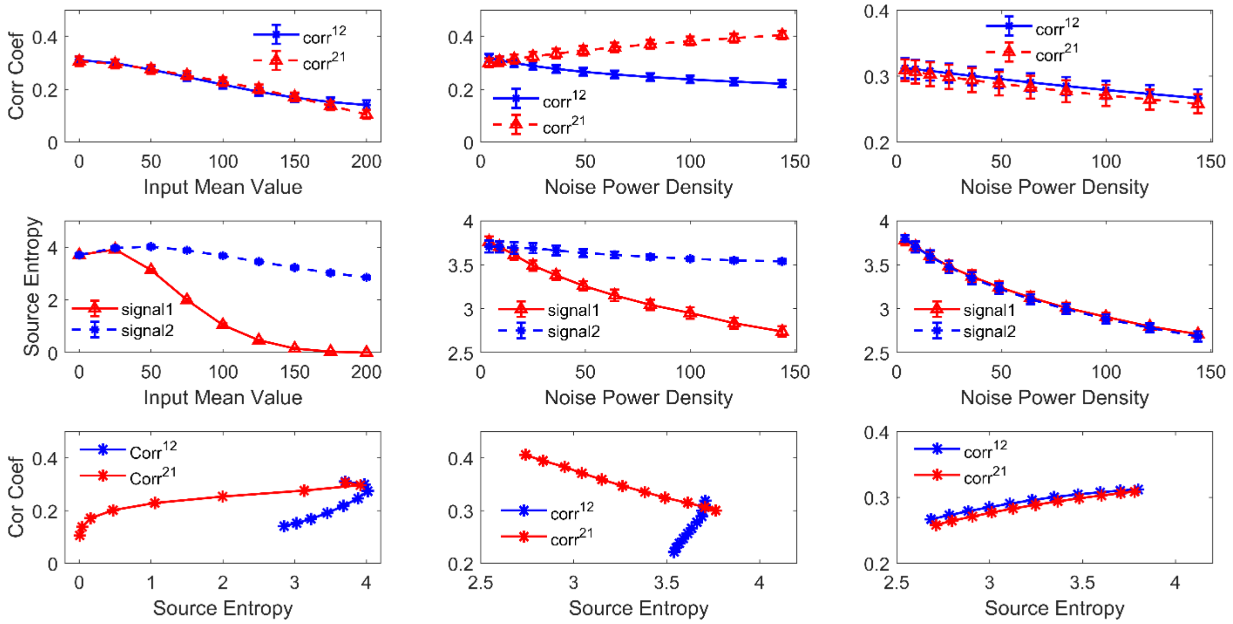

Figure 8S – Effect of the mean value and standard deviation of the input noise on the estimation of DCC. The simulations were performed using two regions connected with excitatory synapses and by varying the mean value m_1 (left panels) and standard deviation σ_1 of noise (middle panels) of the input to ROI1. Finally, the right panels show the case when noise standard deviation was increased in both populations altogether (both parameters σ_1.and σ_2). The first row shows DCC ± SD, while the second row shows entropy (± SD) of the two signals, vs. the input values. Finally, the third row plots DCC vs. the entropy of the source signal See text for more details.

Figure 9S

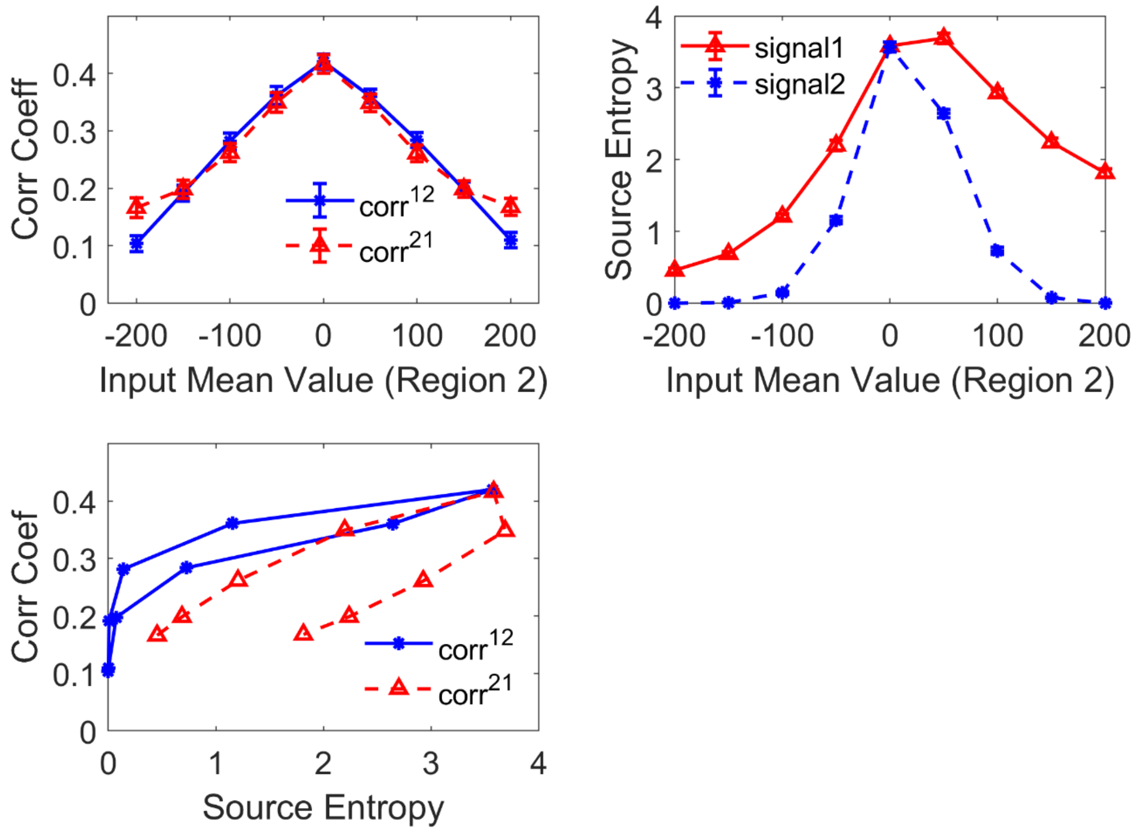

Figure 9S – Effect of the region working point on the estimation of DCC. The simulations were performed using two regions connected with excitatory synapses and by varying the input mean value to region 2. The meaning of the plots is the same as in Fig. 8S. See also the text for more details.

Figure 10S

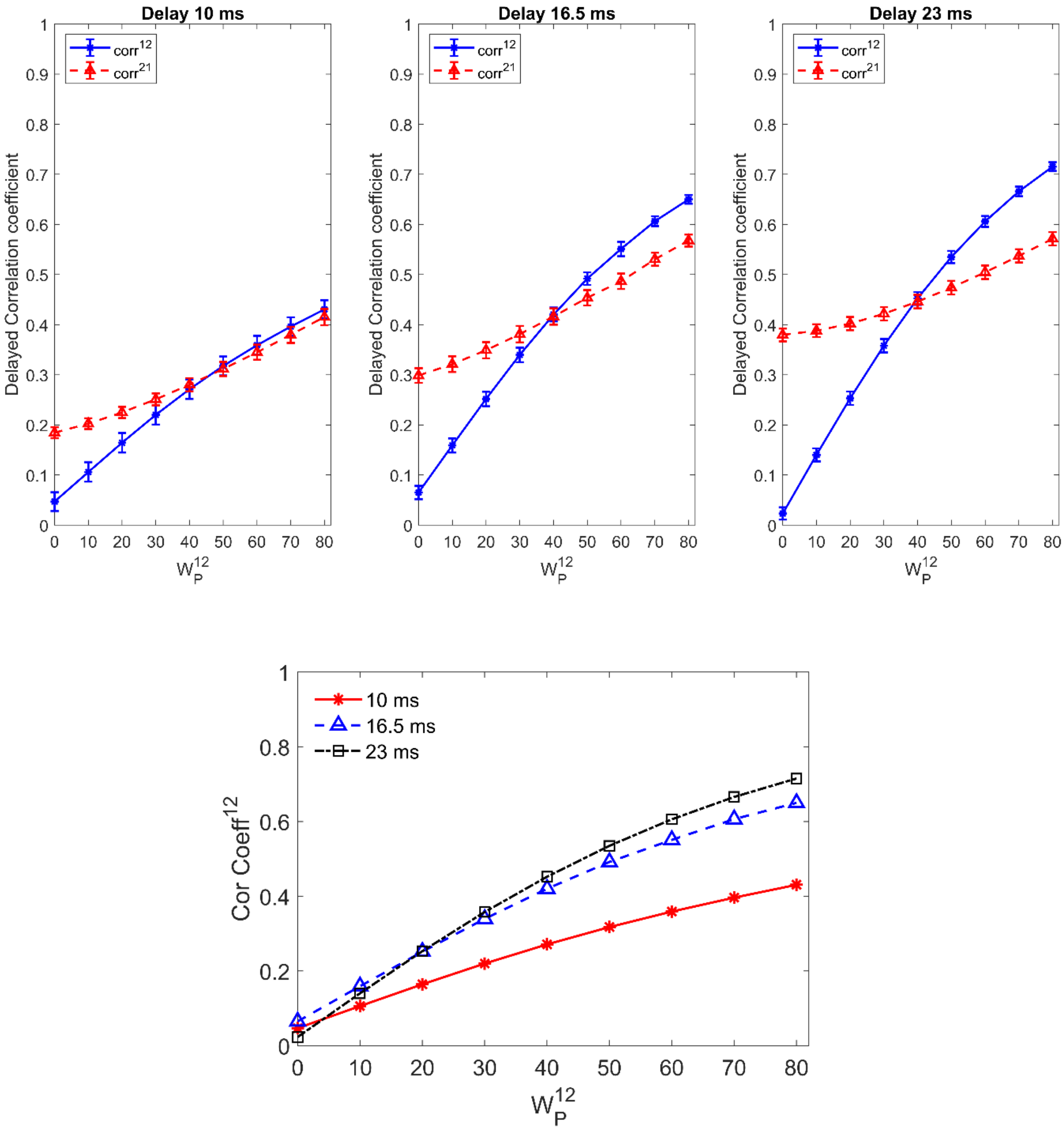

Figure 10S – Effect of the delay between the two regions on the estimation of DCC. The simulations were performed using two regions connected with excitatory synapses. The simulations were repeated with different delays. See the text for more details.

Figure 11S

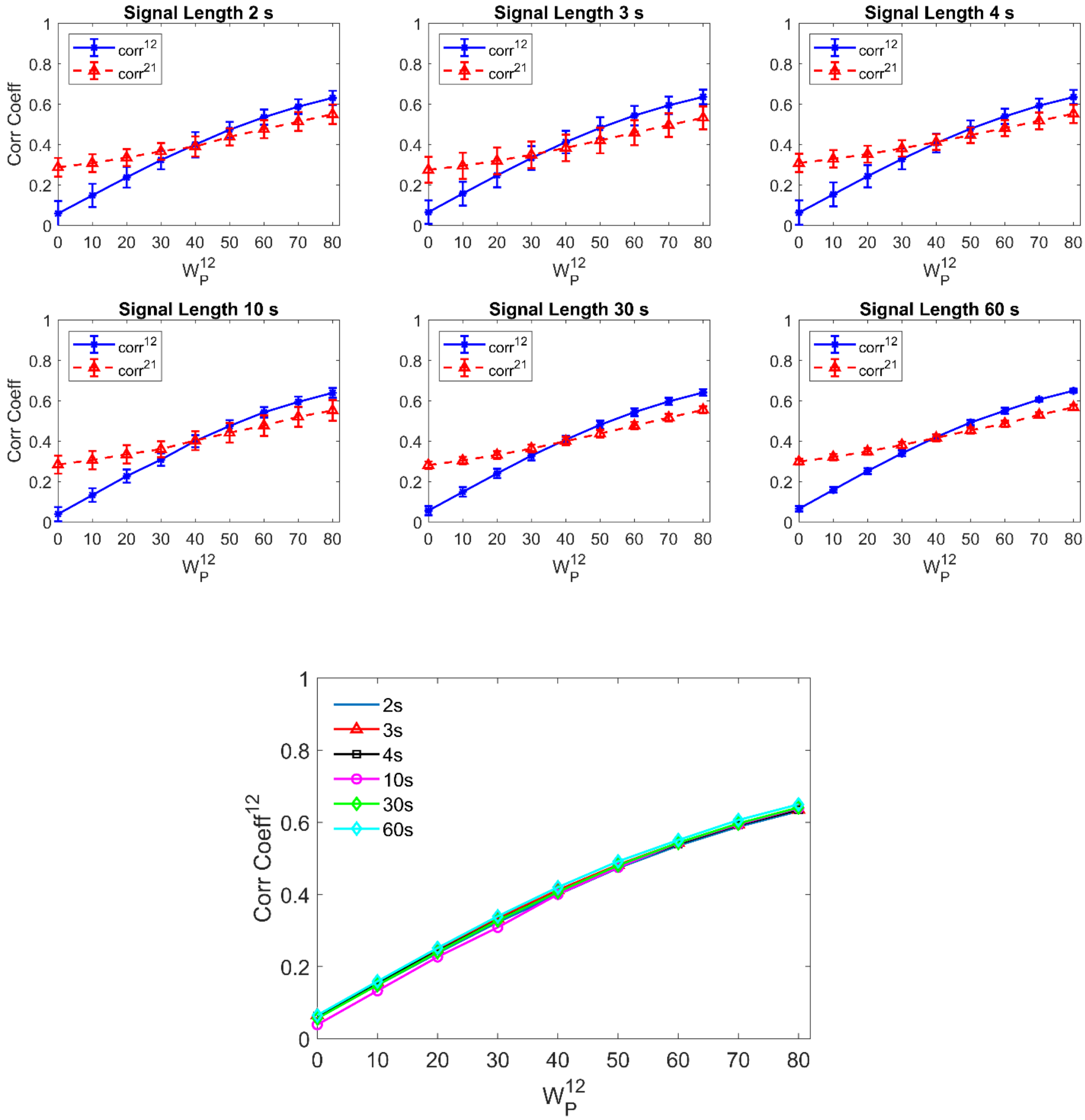

Figure 11S – Effect of the duration of the signal on the estimation of DCC. The simulations were performed using two regions connected with excitatory synapses. The simulations were repeated with different durations of the signals. See the text for more details.
